# Myotubularin-related protein 7 inhibits insulin signaling in colorectal cancer

**DOI:** 10.18632/oncotarget.10466

**Published:** 2016-07-07

**Authors:** Philip Weidner, Michaela Söhn, Tobias Gutting, Teresa Friedrich, Timo Gaiser, Julia Magdeburg, Peter Kienle, Hermelindis Ruh, Carsten Hopf, Hans-Michael Behrens, Christoph Röcken, Tamar Hanoch, Rony Seger, Matthias P.A. Ebert, Elke Burgermeister

**Affiliations:** ^1^ Department of Medicine II, Universitätsmedizin Mannheim, Medical Faculty Mannheim, Heidelberg University, D-68167 Mannheim, Germany; ^2^ Institute of Pathology, Universitätsmedizin Mannheim, Medical Faculty Mannheim, Heidelberg University, D-68167 Mannheim, Germany; ^3^ Department of Surgery, Universitätsmedizin Mannheim, Medical Faculty Mannheim, Heidelberg University, D-68167 Mannheim, Germany; ^4^ ABIMAS Research Center, Mannheim University of Applied Sciences, D-68163 Mannheim, Germany; ^5^ Institute of Pathology, Christian Albrecht University, D-24105 Kiel, Germany; ^6^ Department of Biological Regulation, Weizmann Institute of Science, I-7610001 Rehovot, Israel

**Keywords:** colorectal cancer, insulin, MTMR7, phosphatase, myotubularin

## Abstract

Phosphoinositide (PIP) phosphatases such as myotubularins (MTMs) inhibit growth factor receptor signaling. However, the function of myotubularin-related protein 7 (MTMR7) in cancer is unknown. We show that MTMR7 protein was down-regulated with increasing tumor grade (G), size (T) and stage (UICC) in patients with colorectal cancer (CRC) (n=1786). The presence of MTMR7 in the stroma correlated with poor prognosis, whereas MTMR7 expression in the tumor was not predictive for patients' survival. Insulin reduced MTMR7 protein levels in human CRC cell lines, and CRC patients with type 2 diabetes mellitus (T2DM) or loss of imprinting (LOI) of insulin-like growth factor 2 (*IGF2)* had an increased risk for MTMR7 loss. Mechanistically, MTMR7 lowered PIPs and inhibited insulin-mediated AKT-ERK1/2 signaling and proliferation in human CRC cell lines. MTMR7 provides a novel link between growth factor signaling and cancer, and may thus constitute a potential marker or drug target for human CRC.

## INTRODUCTION

Chronic or constitutive stimulation of receptors for insulin, insulin-like (IGF) and epidermal (EGF) growth factors activates RAS-ERK1/2 and PI3K-AKT-mTOR signaling [1] [2]. Increased activity of those proteins involved in signal transduction and secretion of ligands drive tumor initiation and progression in colorectal cancer (CRC) [3]. Thereby, hyperinsulinemia may contribute to an increased cancer risk in patients with type 2 diabetes mellitus (T2DM) [4–6]. However, the molecular pathways which link metabolism and cancer are unknown. Activating mutations or amplifications in genes of growth factor receptor tyrosine kinases (RTKs) are common in tumors [1] [2] and underlie non-response to current clinical therapies [7]. Thus, new drugable targets that inhibit RTK signaling are needed [8].

We identified myotubularin-related protein 7 (MTMR7) [9], a member of the myotubularin (MTM) lipid phosphatase family [10], as an inhibitor of insulin signaling. MTMs consist of N-terminal plextrin homology (PH), central protein tyrosine phosphatase (PTP) and C-terminal SET-interaction (SID) and coiled coiled (CC) domains [11]. Heterodimers are formed between catalytically active and inactive enzymes, e.g. MTMR6/7/8 with MTMR9 [12], which dephosphorylate phosphatidyl-inositol-3-monophosphate (PI(3)P) and −3,5-bisphosphate (PI(3,5)P2) at the 3′-position of inositol. MTMs localize to endosomal-lysosomal membranes, initiate vesicle trafficking and autophagy [10] and are mutated in human congenital neuromuscular diseases (such as MTM1) [11]. MTMR7 is also present in a soluble form in the cytoplasm using free inositol-1,3-bisphosphate (Ins(1,3)P2) as a substrate [9]. MTMR7 was detected in brain, muscle, liver and kidney [9], whereas its expression and function in the colorectum is unknown.

We show that MTMR7 inhibited insulin-mediated activation of AKT and ERK1/2 signaling and reduced proliferation of human CRC cells. MTMR7 protein was down-regulated in human CRC cells and patient tissues. Expression of MTMR7 in the tumor was independent of prognosis, whereas stromal MTMR7 predicted poor survival in CRC patients. Thus, MTMR7 may constitute a potential target or marker for CRC and a possible link in the cross-talk of lipid phosphatases and growth factor signaling.

## RESULTS

### Expression of MTMR7 in intestinal tissues and cell lines

**Figure 1:**
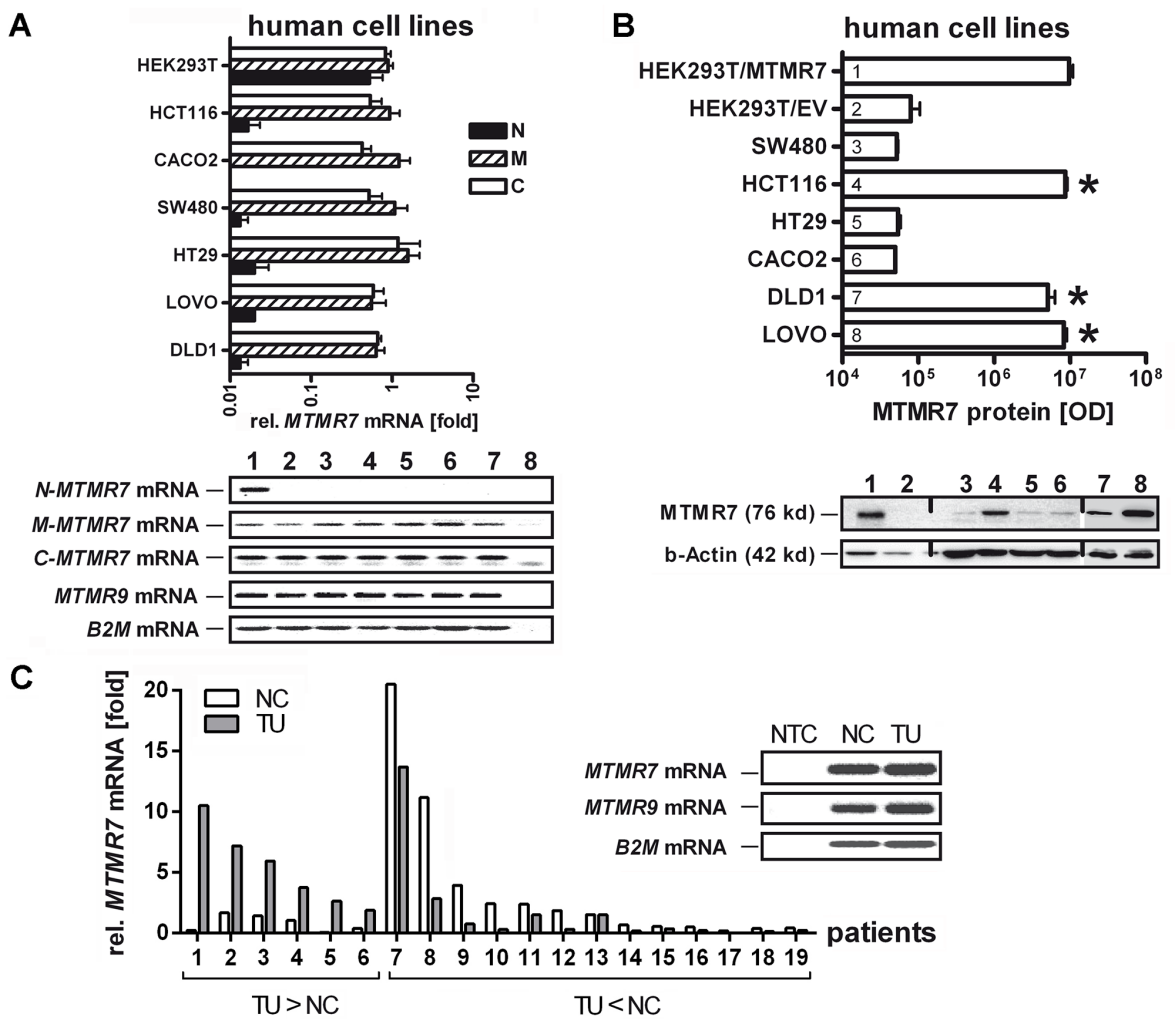
Expression of MTMR7 in human CRC tissues and cell lines **A.** Detection of *MTMR7* cDNA in total RNA extracted from human cell lines. Primers were designed for N-terminal (*N-MTMR7*), central/middle (*M-MTMR7*) and C-terminal (*C-MTMR7*) regions of the CDS as detailed in Supplementary Table S7. Representative agarose gels (endpoint 40 x cycles) are shown together with the quantitative analyses. CT-values from RT-qPCRs on total RNA were normalized to beta2-microglobulin (*B2M*) and calculated as -fold ± S.E. (n=3 per cell line; *p<0.05 *vs*. HT29; Kruskal Wallis test). Legend: 1 = HEK293T, 2 = HCT116, 3 = Caco2, 4 = SW480, 5 = HT29, 6 = LOVO, 7 = DLD1, 8 = NTC = non-template (water) control without specific bands. The weak band below contains primer dimers (size <50 bp). Expected sizes of amplification products: *N-MTMR7 =* 345 bp, *M-MTMR7* = 134 bp, *C-MTMR7* = 131 bp, *MTMR9* = 174 bp, *B2M* = 85 bp. **B.** Detection of 76 kDa MTMR7 protein. Western blots from total cell lysates using the MTMR7 Ab (Abcam) are shown together with the quantitative analyses. HEK293T cells were transiently transfected with pT-MTMR7 and empty vector (EV) expression plasmids for 48 h. Tansfected cells were used as positive (HEK293T/MTMR7) and negative (HEK293T/EV) controls. OD values of bands in gels were normalized to beta-actin and calculated as means ± S.E. (n=3 per cell line; *p<0.05 *vs*. EV; Kruskal Wallis test). Legend: 1 = HEK293T/MTMR7, 2 = HEK293T/EV, 3 = SW480, 4 = HCT116, 5 = HT29, 6 = Caco2, 7 = DLD1, 8 = LOVO. **C.** Detection of *MTMR7* mRNA in CRC. Quantification was done in total RNA from frozen human tumor (TU) compared to matched normal colon (NC) tissues (both with mixed tumor and stroma cells). CT-values from RT-qPCRs normalized to *B2M* were calculated as -fold (n=19 cases; p=0.052; n=6 with up-regulation TU>NC *vs.* n=13 with down-regulation TU<NC; Fisher Exact test). Insert: Representative bands from RT-PCRs in agarose gels detecting *MTMR7* (134 bp) and *MTMR9* (174 bp) mRNAs (endpoint 40 x cycles). NTC = non-template (water) control.

Three different primer sets were designed to amplify the N- and C-terminal ends and the central (mid) region of the full-length (FL) *MTMR7* cDNA (Figure 1A). RT-qPCR analyses showed that *MTMR7* mRNA was present in six human CRC cell lines (SW480, HCT116, Caco2, HT29, LOVO, DLD1) and in non-cancer HEK293T cells. We then PCR-amplified and inserted FL *MTMR7* cDNA from HCT116 cells into pTarget (pT) expression vector. HEK293T cells were transiently transfected with pT-MTMR7 plasmid, and total cell lysate (TCL) expressing the ectopic 76 kDa protein was used as a positive control for validation of MTMR7 Ab (from Abcam) compared to negative control cells transfected with empty vector (EV). Western blot analyses (Figure 1B) demonstrated expression of endogenous 76 kDa MTMR7 protein in a subset of CRC cell lines (HCT116, LOVO, DLD1). Transformed (AGS, PATU8902) and non-cancer cell lines of different tumor types or species (e.g. COS7) were positive as well (not shown). Abs specific for the C-terminal part of MTMR7 recognized an additional band at 54 kDa (unpublished observation), indicating that protein isoforms or mRNA variants may exist as described for mice [9]. *MTMR7* mRNA was also detected in frozen samples of normal colon (NC) and tumor (TU) tissues from CRC patients (Figure 1C) and in mouse organs (Supplementary Figure S1). RT-qPCR analyses revealed coexpression of *MTMR9/Mtmr9* mRNAs with *MTMR7/Mtmr7* in all tissues and cell lines examined. *MTMR7* mRNA was down-regulated in 63 % of CRC samples compared to matched normal colon tissue (n=19 cases), indicative of a loss of expression in a subset of tumors.

### Genetic alterations of the *MTMR7* gene in human CRC

**Figure 2:**
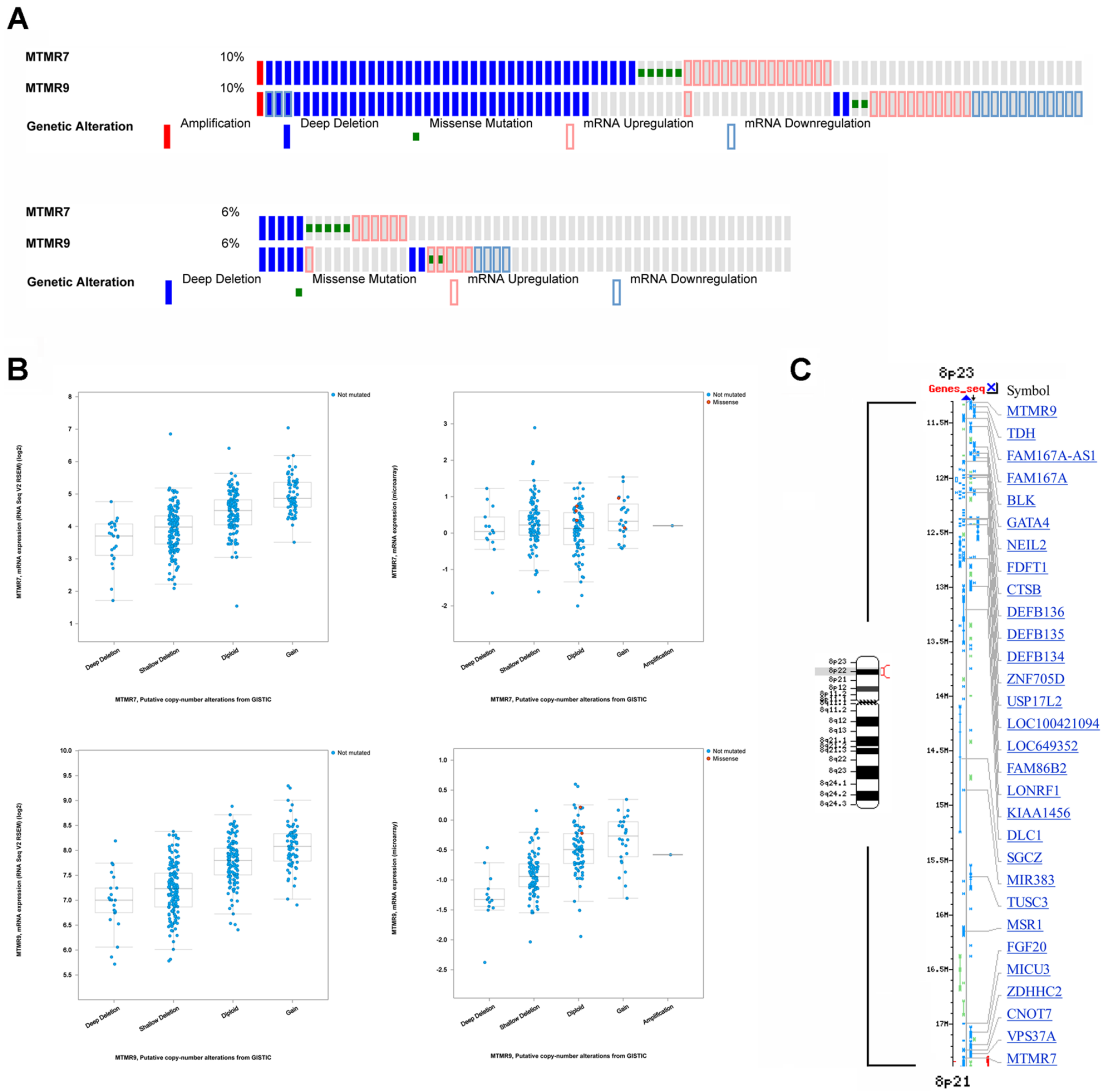
Genomic alterations in human CRC **A**. Co-occurrence of changes (mainly deletions and mRNA alterations) in human *MTMR7* and *MTMR9* genes. Data were retrieved as Oncoprint® files from the webportal cBioportal of Cancer Genomics based on two data sets for CRC patients. Top panel: colorectal carcinoma TCGA_Provisional (n=631 cases); Bottom panel: colorectal carcinoma TCGA_Nature 2012 (n=195 cases). Note that each patient is represented by a bar. Only patient cases with alterations are depicted, whereas cases without alterations appear in grey or are cut off at the right end of the bar plot. The percentage (%) of altered cases compared to the total case number is shown on the left end of the bar plots. **B.** Correlation of mRNA expression and genomic alterations in *MTMR7* and *MTMR9* genes. Data were retrieved from the webportal cBioportal of Cancer Genomics as in (A). The mRNA expression (RNAseq V2 RSEM) (log2) is plotted against putative copy number alterations (CNA) from GISTIC. **C.** Chromosomal localization of *MTMR7* and *MTMR9* genes. Gene order and symbols for the genomic regions on chromosome 8p21-23 (red bracket) are depicted using NCBI Mapviewer. The small arm of chromosome 8 is frequently rearranged in human cancers and comprises tumor suppressor and oncogenes as indicated by gene symbols. Legend: M = Megabase pairs.

*In silico* screening of a large series of CRC patients using the cBioportal of Cancer Genomics [13, 14] was conducted on the data sets: TCGA_Provisional (n=631 cases) and TCGA_Nature2012 (n=195 cases) [3]. The *MTMR7* gene was altered in 10%, *MTMR9* in 6% of patient cases (n=826) (Figure 2A). Mining of the human cancer cell line encyclopedia (CCLE) data base evinced alterations of *MTMR7* in 11% and of *MTMR9* in 16% of cell lines (n=1019) [15] (data not shown). The overall alteration rate of *MTMR7* and *MTMR9* genes was <10% in 86 cancer studies from >15 different tumor entities, indicating that the two genes are of importance beyond CRC (Supplementary Table S1 and S2). In CRC, alterations were mainly due to genomic deletions and changes in mRNA expression levels, whereas the somatic mutation rate was low (<1%). The down-regulation of mRNA correlated with copy number alterations (CNAs) for both genes (Figure 2B) and patient data sets (Supplementary Figure S2). A strong co-occurrence of alterations in *MTMR7* and *MTMR9* genes was observed in human CRC (n=631 cases; Odds ratio >3, *p<0.001, Fisher Exact test) and the CCLE (n=1019; Odds ratio 1.552, *p<0.001, Fisher Exact test), consistent with the biochemical evidence that MTMR7 forms heterodimers with MTMR9 [9]. Both genes colocalized to the short arm of chromosome 8 (8p21-p23) which is frequently subjected to genomic rearrangements in cancers [16, 17] (Figure 2C), providing an underlying structural basis for the alterations observed in the cBioportal data sets.

### Loss of MTMR7 protein is a common event in human CRC

To visualize expression of MTMR7 protein *in situ*, we performed immunohistochemistry (IHC) on normal human colon tissue with the Ab (from Abcam) used for Western blotting (Supplementary Figure S3). MTMR7 staining was found to be weak in the cytoplasm of normal intestinal epithelial cells, but was most prominent in smooth muscle cells of the muscularis mucosae and in perivascular smooth muscle cells of the submucosa. Single MTMR7 positive stromal cells in the lamina propria partially colocalized with alpha-smooth muscle actin and macrophages (data not shown).

**Figure 3:**
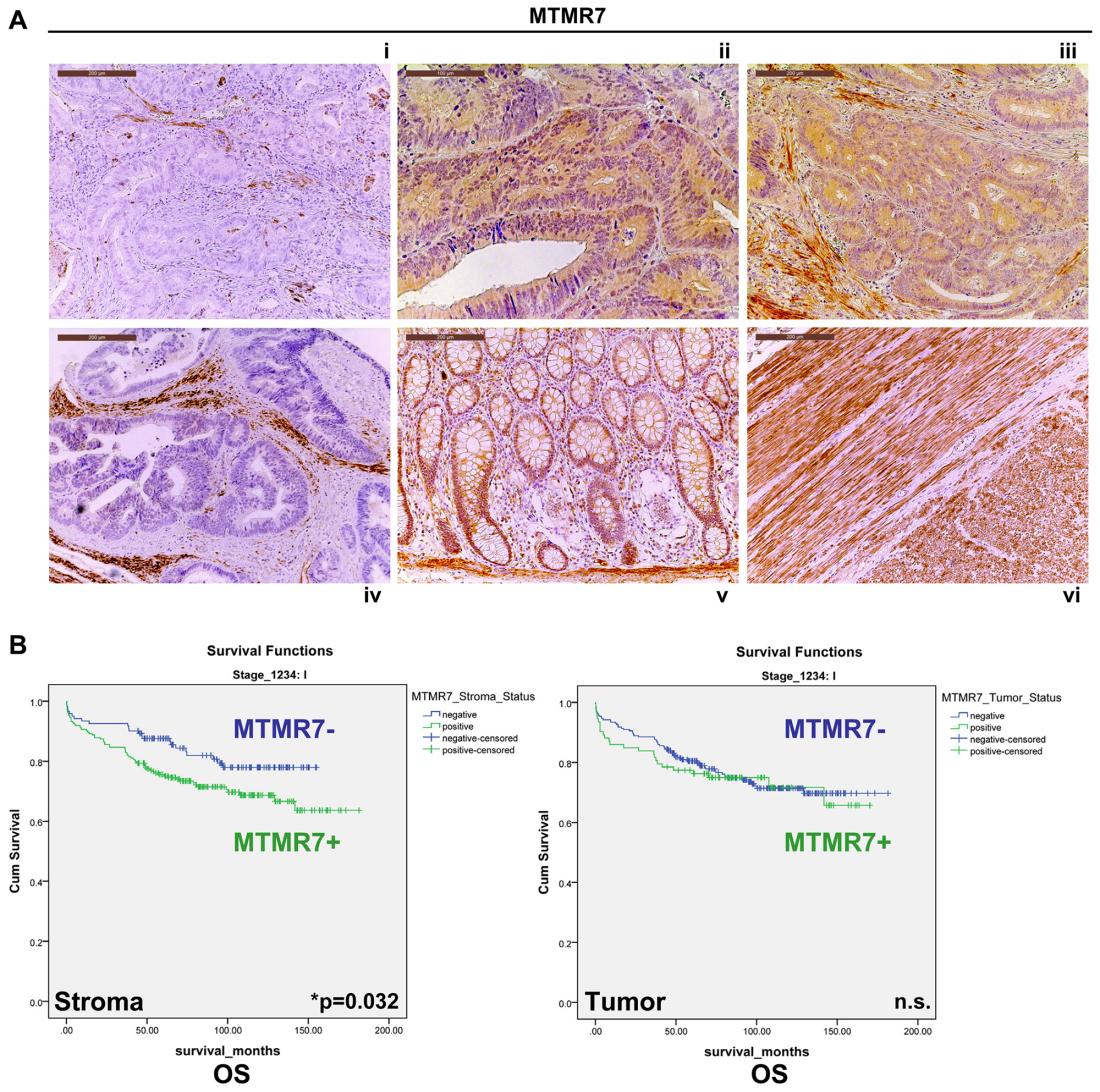
Expression and prognostic significance of MTMR7 in CRC patients **A.** Immunohistochemistry (IHC) with MTMR7 Ab (from Abcam) on tissue microarrays (TMAs) with tumor (n=1776) specimens from CRC patients. Representative images are shown. MTMR7 (brown color) was localized in the cytoplasm. MTMR7 staining was absent (i) or present (ii, iii) in a subset of tumors or adjacent stroma cells (i, iv). MTMR7 (positive control) was also detectable in enterocytes and the lamina propria of the non-malignant colon (v) and in smooth muscle tissue (vi). Original magnifications 100x and 200x. **B.** Stromal expression of MTMR7 is a negative predictor of overall (OS) and tumor-specific (TSS) survival in CRC patients with UICC stage 1 tumors. Dichotome analysis of MTMR7 protein expression in the tumor (n=1776) and adjacent stroma (n=1786) tissue in TMAs from CRC patients as described in A. MTMR7 positivity was associated to survival proportions in months. Kaplan-Meier curves are presented for OS only (*p=0.032; n=345 cases in UICC stage 1; MTMR7 positive *vs.* negative; log rank test). The complete data sets for OS and TSS in UICC stages I-IV are shown in Supplementary Table S4.

MTMR7 was present in human CRC tumor cells or completely absent. To explore the clinical significance of this dichotome MTMR7 expression, tissue microarrays (TMAs) with tumor and adjacent stroma tissue of a large CRC patient cohort were analysed (Figure 3A). Univariate statistical evaluation of the stainings evinced an inverse relation between MTMR7 positivity and clinical parameters (Supplementary Figure S4, Supplementary Table S3). Consistent with the findings on *MTMR7* mRNA expression, MTMR7 protein was lost in 77.5 % (n=1776) of tumor and 35.4 % (n=1786) of stroma samples. There was no correlation between MTMR7 positivity and patient age or gender. However, MTMR7 expression was higher in the distal colon (sigmoid and rectum) than in the proximal colon (coecum and ascending colon) both in tumor and stroma (*p<0.001). MTMR7 positivity in tumor cells was negatively associated with local tumor growth (T1/2 *vs*. T3/4: *p=0.003), tumor stage (UICC I-IV: *p=0.027) and grade (G: *p=0.015). These data indicated that MTMR7 is lost during dedifferentiation and invasive growth of the tumor, consistent with a putative role for MTMR7 as a tumor suppressor in the colon.

### MTMR7 protein in the stroma is a negative predictor of CRC patient survival

Regarding prognostic relevance, we found that MTMR7 positivity in the stroma tissue surrounding the tumor cells was associated with a reduced 5- and 10-year survival rate for patients with CRC in the UICC stage 1 (Figure 3B). This held true for overall survival (OS) (5-year: MTMR7 stroma negative 88 % *vs*. 76 % positive; 10-year: MTMR7 stroma negative 78 % *vs*. 69 % positive) and tumor-specific survival (TSS) (5-year: MTMR7 stroma negative 96 % *vs.* 88 % positive; 10-year: MTMR7 stroma negative 93 % *vs.* 86 % positive) (n=345 cases, *p=0.032, log rank test). However, there was no association between MTMR7 protein expression in the tumor and survival at any UICC stage (Supplementary Table S4). *In silico* screening of additional data sets (TCGA_Nature, _Cell, _Provisional [3]) using cBioportal [13, 14] revealed that *MTMR7* (but not *MTMR9*) was of prognostic value in other tumor entities including liver, pancreas, head and neck or renal cancer. However, conclusions were limited due to low case numbers (Supplementary Table S5).

### MTMR7 is lost in CRC under conditions of active insulin/IGF2 signaling

**Figure 4:**
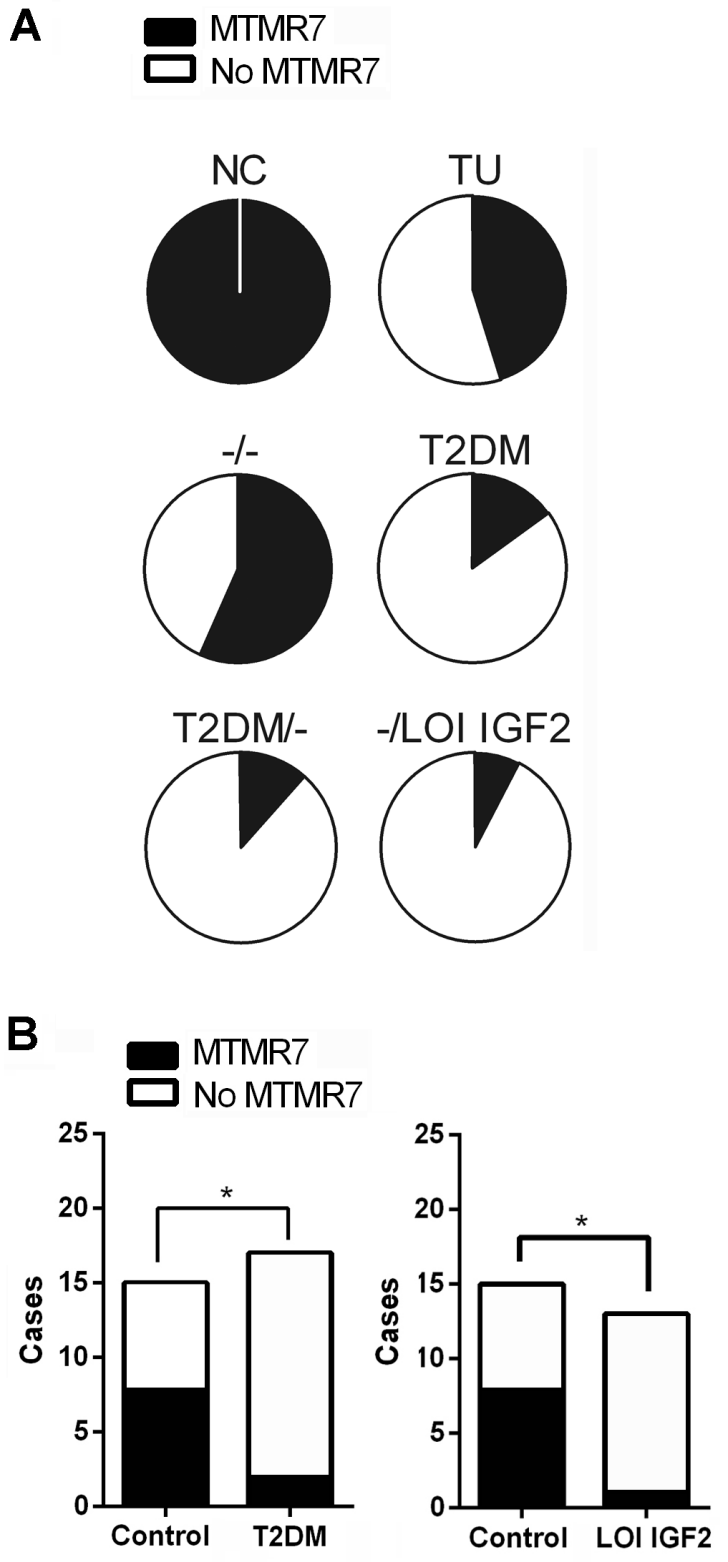
Loss of MTMR7 protein is a common event in human CRC **A.** Dichotome analysis of MTMR7 protein expression in IHC stainings from a second independent patient cohort with known metabolic parameters. The intensity and frequency of MTMR7 staining (Abcam Ab) in normal colon (NC) and CRC tumor (TU) tissue (n=113 total cases) was calculated as % negative (white = MTMR7 absent) *vs.* positive (black = MTMR7 present) cases. Control group −/− = cases with neither T2DM nor LOI *IGF2* (for details see Supplementary Table S6). **B.** MTMR7 is preferentially lost in tumors of patients with T2DM or *IGF2* LOI. Dichotome analysis of MTMR7 positivity in IHC images of the cohort shown in A. MTMR7 expression in CRC tumor tissue was assessed in patient cases without (control) or with T2DM or *IGF2* LOI. The intensity and frequency of MTMR7 staining in tumor tissues are expressed as negative *vs.* positive cases (*p<0.05 LOI or T2DM *vs.* control; Fisher Exact test).

To identify factors which influence MTMR7 expression, we analysed a second independent cohort of CRC patients with metabolic parameters known to activate RTK signaling: type 2 diabetes mellitus (T2DM) and loss of imprinting (LOI) of the *IGF2* gene in the tumor. The collection consisted of CRC tumor samples (n=113), normal colon biopsies of healthy individuals (n=8) and normal colon tissue adjacent to the tumor site of T2DM patients (n=9). While detectable in all samples of benign colon tissue (n=17), MTMR7 staining was absent in 55% (62 of 113) of the CRC samples (Figure 4A, Supplementary Table S6). The negative correlation between MTMR7 positivity and tumor grade was confirmed: 64 % (7 of 11) of G1 tumors were positive for MTMR7, in contrast to 47 % (33 of 70) of G2 tumors and 32 % (8 of 25) of G3 tumors. There was no correlation between MTMR7 and additional tumor specifications or patient characteristics such as *KRAS* mutations or the body mass index (data not shown). We then separately analysed samples of CRC cases with T2DM (n=17) or *IGF2* LOI (n=13). MTMR7 was lost to a higher percentage in tumor tissues of both patient groups (T2DM: 88%, 15 of 17; *IGF2* LOI: 92%, 12 of 13) than in individuals with neither T2DM nor *IGF2* LOI (44%, 7 of 16) (Figure 4B). *IGF2* LOI (n=13) was associated with a higher risk for loss of MTMR7 in the tumor (OR: 13.71, *p= 0.0157) compared with the control group of CRC cases with neither *IGF2* LOI nor T2DM (n=16). A similar association (OR: 8.57,*p= 0.0209) was stated for CRC patients with T2DM (n=17). MTMR7 loss was exclusively detected in specimens of malignant tissue. There was no case of reduced MTMR7 expression in the benign colonic mucosa, neither in cases of T2DM nor in healthy individuals. Thus, metabolic factors which activate RTK signaling positively correlate with loss of MTMR7 in human CRC.

### MTMR7 decreases cellular PI(3)P levels

**Figure 5:**
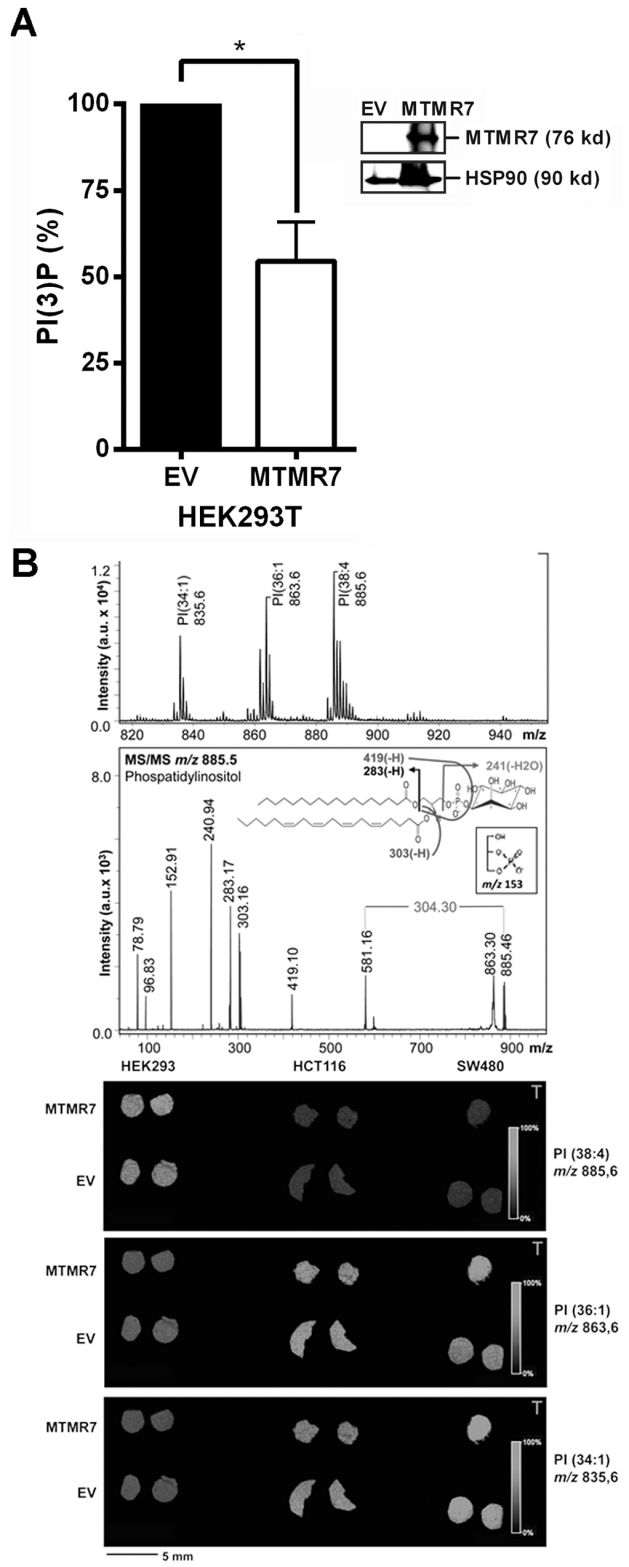
MTMR7 decreases cellular PI(3)P **A.** PI(3)P ELISA. HEK293T cells were transfected with EV or MTMR7 plasmids for 48 h. PI(3)P was quantified in extracted lipids. OD values from ELISA were pmol/10^6^ cells and calculated as % ± S.E. (n=4; *p=0.016 MTMR7 *vs.* EV; Wilcoxon signed rank test) compared to EV controls. Insert: Western blot from solvent-precipitated proteins after lipid-extraction confirming overexpression of MTMR7 compared with EV and HSP90 loading control. **B.** PI MALDI-IMS. HEK293T, HCT116 and SW480 cells were transiently transfected with EV or MTMR7 plasmids, and harvested after 48 h. Lipid mass images were collected from cryosections of frozen cell pellets. Top: Mass spectra with Ph-CCA-amide matrix in negative ion mode. MS fragmentation pattern was consistent with PI isoform PI(18:0, 20:4); Bottom: Mass images from PI species with different saturation and length of the carbon side chains. Color-coded intensity bars for PI mass peaks are depicted together with circular cross-sections of frozen cell pellets on matrix-sprayed glass slides.

To elucidate the function of MTMR7 in cells, we measured the enzymatic activity of the phosphatase by ELISA. HEK293T cells were transiently transfected with EV or MTMR7 plasmid for 48 h, and overexpression of 76 kDa MTMR7 protein was confirmed by Western blot (Figure 5A). PIPs were quantified in total acidic lipids extracted from cells after precipitation of the proteins. The amount of mono-phosphorylated PI(3)P was reduced in MTMR7-transfected cells to 54±16 % (n=4, *p=0.016, Wilcoxon signed rank test) compared with EV controls (MTMR7 106±29 *vs.* EV 230±78 pmol/10^6^ cells) (Figure 5A). Similar results were obtained from SW480 and HCT116 cells (data not shown). Levels of PI(3,4,5)P3 were not altered (not shown), corroborating the specificity of MTMR7 towards PI(3)P. MALDI-MS imaging of frozen cell pellets revealed no changes in PI ion intensity, irrespective of the type of fatty acid side chain attached to the glycerol moiety, either (Figure 5B). These data confirmed the enzymatic activity and substrate specificity of MTMR7 and allowed us to explore the function of this phosphatase in human CRC cells.

### MTMR7 inhibits insulin-mediated AKT and ERK1/2 signaling in human CRC cells

**Figure 6:**
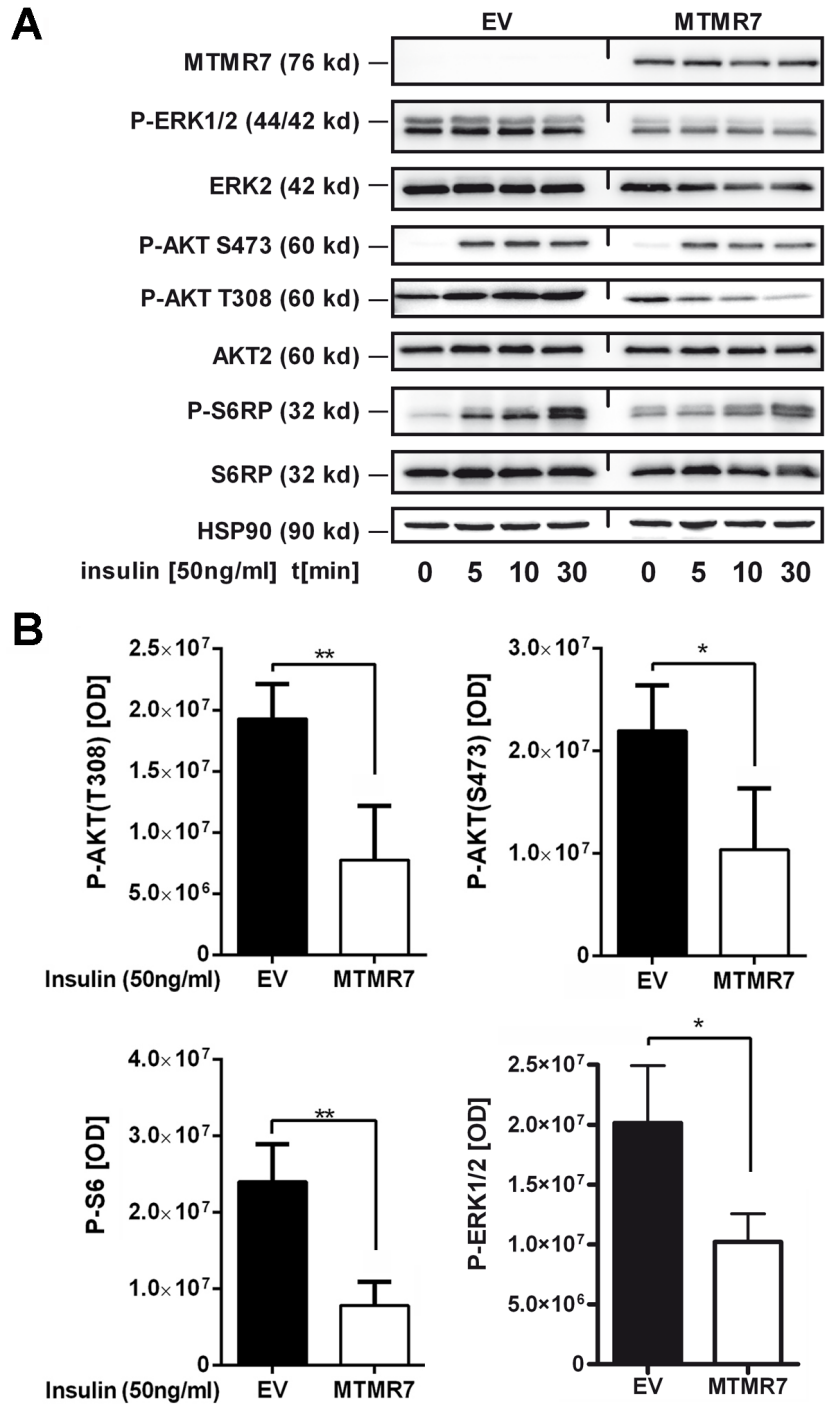
MTMR7 inhibits cellular RTK-signaling Detection of insulin-mediated AKT and ERK1/2 pathway activation. HEK293T, SW480 and HCT116 cells were transiently transfected with EV or MTMR7 plasmids for 48 h, followed by serum-deprival for 24 h, and were then stimulated with insulin (50 ng/ml) for 0 to 30 min. Representative Western blots (**A**) and quantitative analyses (**B**) are shown. OD values from bands in gels are means ± S.E. (n≥3 per cell line; *p<0.05 MTMR7 *vs.* EV; t-test).

Since PIPs activate RTK signaling [2], we tested whether the PIP phosphatase MTMR7 is able to inhibit RTK signaling. Two exemplary down-stream signaling pathways, the AKT-mTOR and ERK1/2 cascades, were studied. HEK293T, SW480 and HCT116 cells were transiently transfected with MTMR7 or EV plasmids for 48 h, followed by serum-deprival for 16 h and subsequent stimulation with insulin (50 ng/ml) for 0 to 30 min. To measure the rapid time course of AKT-mTOR-S6K-S6RP pathway activation, two phosphorylation sites on AKT1/2 were analysed, the THR308 residue, which is the target of PI3K, PIPs and PDK, and the SER473 residue, which is addressed by a feed forward loop through the mTORC2 complex [18]. Western blot analyses (Figure 6A) showed that insulin increased phosphorylation at both AKT sites. Overexpression of MTMR7 reduced phosphorylation on both residues compared to EV controls (S473: EV 2.2±0.2 *vs.* MTMR7 1.0±0.3 *10^7^, *p=0.0206, n=4; T308: EV 1.9±0.1 *vs.* MTMR7 0.8±0.2 *10^7^, *p=0.0046, n=4; t-test). Phosphorylation of the S6 ribosomal protein (S6RP) by S6 kinase (S6K), a down-stream target of the AKT-mTOR pathway, was also lowered (EV 2.4±0.2 *vs.* MTMR7 0.8±0.2 *10^7^, *p=0.0015, n=4, t-test). MTMR7 also diminished insulin-mediated phosphorylation of ERK1/2 (p42/p44) (EV 2.0±0.5 *vs.* MTMR7 1.0±0.2 *10^7^, n=5, *p=0.0397, t-test) (Figure 6B).

In contrast, knock-down of endogenous MTMR7 protein (by ~65%) in HCT116 cells upon transfection of siRNA oligonucleotides augmented phosphorylation of ERK1/2 triggered by the ERK1/2-pathway activators tetraphorbolacetate (TPA) (Supplementary Figure S5) or EGF (not shown) compared to cells which received scrambled control siRNA (si MTMR7 4.9±0.5 *vs*. si Control 3.2±0.3, n=3, *p=0.012, t-test). These data demonstrated that MTMR7 inhibits the AKT-mTOR and RAS-ERK1/2 signaling cascades.

### MTMR7 reduces proliferation of human CRC cell lines

**Figure 7:**
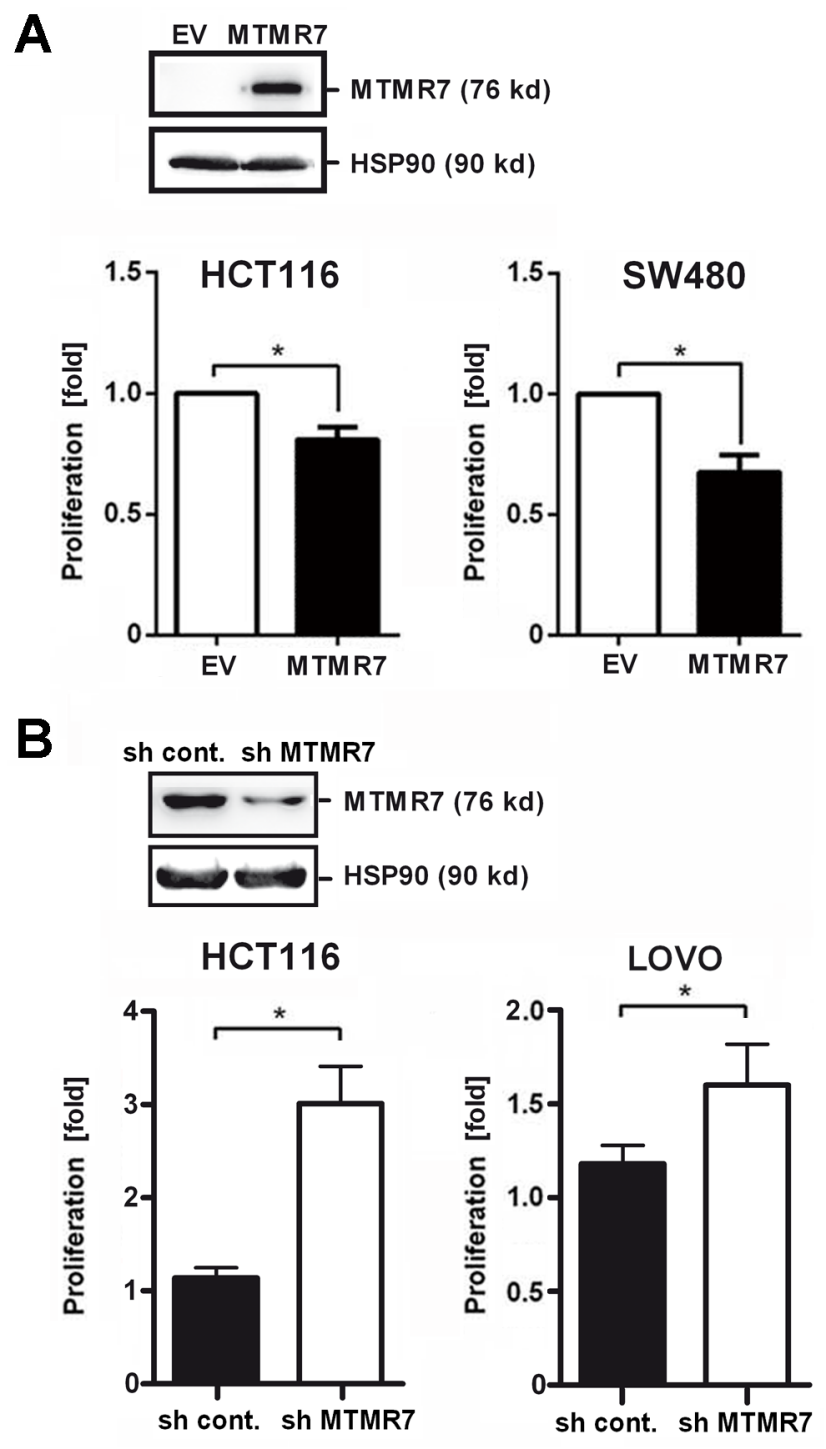
MTMR7 inhibits cell growth **A.** MTMR7 overexpression reduces proliferation. HCT116 and SW480 cell clones stably transfected with MTMR7 or EV plasmid were subjected to MTT proliferation assay after 5 days of adherent growth. OD values were calculated as -fold ± S.E. (n=3 clones; *p<0.05 MTMR7 *vs.* EV; t-test) compared with day 0 as detailed in the methods section. Insert: Western blot confirming overexpression of MTMR7 protein compared with EV and HSP90 loading control. **B.** MTMR7 knock-down accelerates proliferation. HCT116 and LOVO cells were transiently transfected with MTMR7- or control-shRNA plasmid for 24 h, and proliferation was measured after 3 days. OD values were calculated as fold ± S.E. (n=3; *p<0.05 MTMR7-shRNA *vs.* control-shRNA; t-test) compared with day 0. Insert: Western blot confirming reduction of MTMR7 protein by shRNA compared with HSP90 loading control.

To assess whether signaling inhibition by MTMR7 translates into a cellular response, cell proliferation was measured. Clonal lines of SW480 and HCT116 cells stably transfected with MTMR7 or EV plasmid were grown for 5 days in standard medium, followed by quantification of cell growth by MTT assay (Figure 7A). MTMR7 overexpressing clones had reduced growth rates compared with cells that received EV. The cell number was 32% lower for SW480 and 19% lower for HCT116 clones over all time points (MTMR7-EV: SW480 −0.3 ± 0.1, *p=0.010; HCT116 −0.2 ± 0.1, *p=0.033; n=3 clones per genotype; t-test).

*Vice versa*, knock-down of endogenous MTMR7 protein in HCT116 cells (by ~52%) upon transfection of MTMR7-shRNA plasmid accelerated cell proliferation compared with cells receiving control shRNA plasmid (sh MTMR7 3.2 ± 0.5 % *vs.* sh Control 1.1 ± 0.2 %, n=3, *p=0.004, t-test) (Figure 7B). Similar results were obtained from LOVO cells (sh MTMR7 1.6 ± 0.2 % *vs.* sh Control 1.2 ± 0.1 %, n=3, p=0.096, t-test). *KRAS*-WT cells (Caco2, HT29, HEK293T) were less sensitive to MTMR7 silencing than *KRAS*-mutant cells (HCT116, LOVO, SW480, DLD1) (Supplementary Figure S6). Thus, loss of endogenous MTMR7 protein resulted in increased tumor cell proliferation, consistent with the observation that MTMR7 was down-regulated during CRC progression in patients.

### MTMR7 protein is down-regulated by insulin in human CRC cell lines

**Figure 8:**
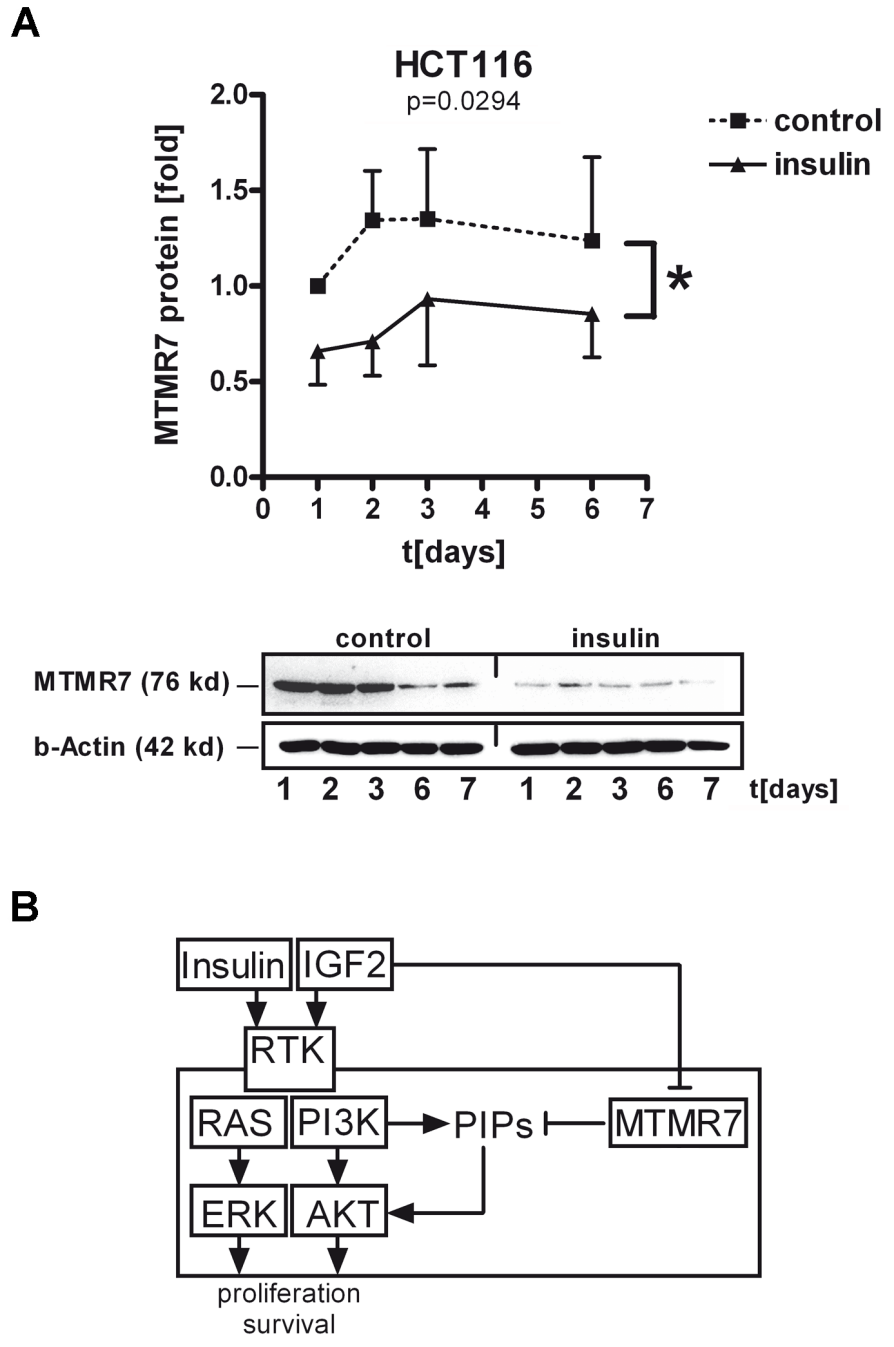
Insulin decreases cellular MTMR7 protein **A.** HCT116 cells were incubated with or without insulin (at 50 ng/ml) in full medium for the times indicated, followed by Western blotting. OD values from MTMR7 bands in gels were calculated as -fold ± S.E. of day 0 in control cells (n=3; *p<0.05 insulin *vs.* control; two-way ANOVA). **B.** Model for MTMR7 function and regulation by insulin. MTMR7 lowers PI3P levels, which results in a decrease of growth factor-mediated AKT/ERK signaling, resulting in inhibition of proliferation of CRC cells. In contrast, insulin itself leads to down-regulation of MTMR7 protein, thus preventing the inhibitory effect of MTMR7 on growth factor signaling.

We finally asked whether long-term exposure of human CRC cells to growth factors reduces MTMR7 protein similar to the observed loss of MTMR7 in CRC patients. To this end, HCT116 cells were grown to 30% confluency and then incubated in full medium (control) or full medium supplemented with insulin (at 50 ng/ml) for 1 to 7 days. Western blot analyses of TCLs demonstrated that MTMR7 protein increased over time in post-confluent cells, consistent with high expression of MTMR7 in terminal differentiated tissues (such as smooth muscle cells). In contrast, MTMR7 protein was decreased in presence of insulin already after 1-2 days compared to controls (insulin 0.79 ± 0.06 *vs.* control 1.23 ± 0.08; n=7; *p=0.0294; two-way ANOVA) (Figure 8A). Similar results were collected from LOVO and DLD1 cells (not shown). In contrast, recombinant growth factors (IGF1, IGF2, EGF), serum shock [20% (v/v) FCS], starvation [0% (v/v) FCS] or other stimuli (rosiglitazone, H_2_O_2_) had no effect (Supplementary Figure S7 and S8). Neither insulin nor any other stimuli tested diminished the expression of *MTMR7* mRNA (data not shown), suggesting a (post)translational mechanism that has to be studied in the future.

Collectively, these data indicated that insulin down-regulates MTMR7 protein as an endogenous inhibitor of growth factor receptor signaling, thereby establishing a potential vicious cycle promoting the proliferation of cancer cells (Figure 8B).

## DISCUSSION

In this study, we described MTMR7 as an inhibitor of insulin signaling in human CRC cells. MTMR7 attenuated two major pathways important for cell proliferation and survival, the AKT and ERK1/2 cascades. This mechanism applied to human cell lines derived from colorectal, gastric, and pancreatic cancer and to embryonic kidney cells, alluding at a more general principle. MTMR7 decreased phosphorylation of ERK1/2 by MEK1/2 on 202/185TEY204/187 and of AKT on S473/T308 residues. Hence, MTMR7 did not block single mTORC1/2 complexes, as described for MTM1 [19, 20] or MTMR3 [21], what would have abrogated phosphorylation exclusively on either SER473, which is part of the AKT-mTORC2 feed forward loop, or THR308, which is targeted by PI3K-PDK1. Instead, MTMR7 inhibited upstream components in these pathways resulting in a decreased production of the lipid second messenger PI(3)P. MTMR8 binds PI3K thereby reducing PI3K activity [22]. Thus, direct inhibition of PI3K by MTMR7 may explain our findings, given the structural homology between MTMR7 and MTMR8 and the similarity in binding of both enzymes to MTMR9 [9, 12]. Inhibition of the AKT pathway by MTMs has been reported for T lymphocytes [23] and myotubes [24, 25] in mice and zebrafish [22]. Thus, our data may contribute to redefine MTMs from “survival” [19, 20, 23, 26] towards “tumor suppressor” phosphatases similar to PTEN [27]. MTMR7 blocked RTK signaling in *KRAS*-WT and *KRAS*-mutated cells, and there was no correlation between MTMR7 loss and *KRAS*-mutations in CRC patients. MTMR7 as a dual AKT-ERK1/2 inhibitor may thus be a possible candidate for novel therapeutical strategies also against *RAS*-mutated tumors [28, 29].

Hyperinsulinemia increases growth factor signaling even if the metabolic function of insulin is disturbed [2]. Peripheral insulin resistance augments IGF1 levels [30], and loss of imprinting (LOI) of the *IGF2* gene is associated with poor survival in CRC [31, 32]. Hence, the patient subgroups in our study represented clinical situations of active growth factor signaling, due to systemic or local secretion of RTK ligands. T2DM and *IGF2* LOI were associated with a higher risk for MTMR7 loss in CRC patients. MTMR7-negative tumors may therefore have a growth advantage under those conditions. MTMR7 was expressed in the benign human colonic tissue of all cases. Thus, loss of MTMR7 did not occur before or during tumor initiation but rather at a later step of tumor progression. Consistent with this observation, insulin also down-regulated MTMR7 protein expression in human CRC cell lines. MTMR7 silencing may thus be a consequence of enhanced growth factor signaling, and its association with CRC progression implicates that MTMR7 restricts or terminates RTK signals.

No prognostic relevance was found for expression of MTMR7 protein in the tumor. Instead, MTMR7 was strongly present in smooth muscle cells of the tumor-stroma. The positive association of stromal MTMR7 with poor survival in UICC stage I CRC patients may implicate tumor-stroma interactions via secreted growth factors [1, 2]. MTMs are activators of autophagy and AMPK signaling [33]. In stroma cells surrounding the tumor with high levels of MTMR7, AMPK may thereby evoke metabolic adaptation to lack of nutrients or oxygen that promotes tumor growth and confers poor prognosis for the patient. More than 65 % of stroma tissue adjacent to the tumor still expressed MTMR7 protein. Thus, future pre/clinical studies have to explore the function of MTMR7 in the tumor microenvironment and its potential as a marker or drug target in human CRC.

## MATERIALS AND METHODS

### Subjects

Histological specimens were obtained by surgical resection of primary tumors or metastases. Samples were collected, histologically classified and stored as formalin-fixed and paraffin-embedded (FFPE) or fresh frozen material. Specimens were provided as tissue microarray (TMA, n=1786, Supplementary Table S3) [34] or whole tissue sections [35, 36]. The patient collective (Supplementary Table S6) with known metabolic parameters (T2DM, LOI *IGF2*) comprised tumor samples of CRC patients (total n=113) and normal colon biopsies from 8 healthy male individuals and 9 tissue samples from normal colon adjacent to the tumor from CRC patients with T2DM (total n=17). The study was approved by the Ethics Committees of the Universities of Heidelberg and Kiel.

### Detection of *IGF2* LOI

Genomic DNA was extracted from fresh frozen CRC and normal mucosa samples using Purigene Kit (Qiagen, Hilden, Germany) and genotyped for a single-nucleotide polymorphism (SNP) (820 A/G, refSNP IDrs680) located in the *IGF2* exon 9 using an allele-specific PCR with primers IGF2rs680F 5′-GAATTGGCTGAGAAACAATTGGC-3′ and IGF2rs680Rt 5′-CCACCTGTGATTTCTGGGGT-3′ specific for the “A” allele und IGF2rs680Rc 5′-CCACCTGTGATTTCTGGGGC-3′ specific for the “G” allele. Beta-globin forward 5′-GGTTGGCCAATCTACTCCCAGG-3′ and reverse 5′-GGTTGGCCAATCTACTCCCAGG-3′ primers were used to control for DNA-quality. The PCR was performed with 95°C for 2 min followed by 10 x cycles (94°C for 20 sec, 65°C for 1 min) and 35 cycles (94°C for 20 sec, 62°C for 1 min and 72°C for 30 sec).

### Reagents

Chemicals were purchased from Merck (Darmstadt, Germany) or Sigma (Heidelberg, Germany). Antibodies were MTMR7 (#121222, Abcam, Cambridge, UK), phospho-AKT-S473 (#4060), phospho-AKT-T308 (#13038), AKT (#9272), phospho-S6 ribosomal protein (#4858), S6 ribosomal protein (#2217), phospho-ERK1/2 (#4370) (all from Cell Signaling, Danvers, MA), ERK2 (sc-154), HSP90 (sc-7947) (both from Santa Cruz Biotech., CA) and beta-actin (A1978, Sigma). Rosiglitazone (rosi) was obtained from Cayman Chemical Company (Ann Arbor, Michigan). 12-O-tetradecanoylphorbol-13-acetate (TPA) and human recombinant IGF1/2 were from Sigma, insulin and EGF from Roche Diagnostics GmbH, Mannheim, Germany.

### Expression plasmids

The human full-length (FL) cDNA of MTMR7 (start codon MEHIRT, aa 1-660, 76 kDa, NM_004686.4) was amplified from total RNA extracted from HEK293T cells and inserted with and without an N-terminal FLAG-tag into pTarget (pT) vector (Promega GmbH, Mannheim, Germany). Empty vector (EV) pT was used as a negative control.

### Cell culture

Human embryonic kidney (HEK293T) and CRC cell lines were from the American Type Culture Collection (ATCC, Rockville, MD). Cell lines were maintained at 37°C in a humidified atmosphere of 5% CO_2_ and 95% air in high-glucose DMEM (#41965) supplemented with 10% (v/v) fetal calf serum (FCS) (#SV30160), 20 mM glutamine and penicillin / streptomycin (1000 units/ml; all from from Thermo Scientific), herewith defined as “full” medium. Stably transfected clones of parental cell lines were maintained as previously described [36]. In brief, HCT116 and SW480 cells were transfected with pT-MTMR7 or empty vector (EV) using Turbofect (Thermo Scientific) for 6 h, followed by a change to full medium and incubation for additional 42 h. Thereafter, transfectants were selected for 3 weeks in full medium supplemented with 500 μg/ml G418 (Thermo Scientific), and single clones were subcultivated for additional 4 weeks at 250 μg/ml G418. Clones were tested for the presence of MTMR7 by RT-qPCR and Western blotting.

For insulin-mediated down-regulation of MTMR7 protein, 200.000 cells/well were seeded into a 6-well plate. After 24 h (at ~30% confluency), growth factors or other agents (rosi, H_2_O_2_) were added to the monolayer in full medium. Alternatively, medium was replaced to full medium with a final concentration of 20% (v/v) FCS, defined as “serum shock”, or cells were kept at 0% (v/v) FCS in DMEM without any supplements, defined as “starvation”. Cells were harvested on a daily basis over a time period of 1-7 days. Untreated (control) cells were kept in a duplicate dish in full medium and were harvested at the same time points as the treated cells. Cells reached confluency after 3 days and were left in the post-confluent state until day 7.

For time course experiments detecting phosphorylated proteins, 1.5×10^6^ cells were seeded per 10 cm dish. The day after, cells were transfected for 6 h before replacement to full medium and incubation for additional 42 h. Cells were then serum-deprived for 16 h in DMEM without any supplements followed by restimulation (with insulin, EGF or TPA) in DMEM without any supplements for 0-60 min before harvesting.

### Gene silencing

SiRNA oligonucleotides were from Dharmacon (SMARTpool: ON-TARGETplus, Thermo Scientific, Lafayette, CO). Cells (500.000/well of a 6-well plate) were transiently transfected for 48 h with a mixture of four MTMR7-specific siRNAs provided as a single reagent or control siRNA using Oligofectamin (Thermo Scientific) as recommended by the manufacturer. ShRNA plasmids were from Qiagen (SureSilencing #KH17308, Hilden, Germany). One control and four separate shRNA designs (MTMR7 NM_004686) were provided and packaged in the same plasmid backbone (pGeneClip, Promega). Cells (500.000/well of a 6-well plate) were transfected with the shRNA-control plasmid or a mixture (1:1:1:1) of the four MTMR7-shRNA plasmids (500 ng/well) using Turbofect for 6 h followed by cultivation in full medium for 1-7 days as suggested by the manufacturer.

### Proliferation assay

Colorimetric cell viability assays were conducted according to the manufacturer's protocols (Roche Diagnostics GmbH, Mannheim, Germany). In brief, 500.000 cells/well were seeded into a 6-well plate. The day after, cells were transfected for 6 h before replacement to full medium and incubation for additional 18 h. Cells were then trypsinized and reseeded at a density of 2000 cells/well of 96-well plates and grown for 1-7 days before adding 3-(4,5-dimethylthiazol-2-yl)-2,5- diphenyltetrazolium bromide (MTT) reagent and measurement of optical density (OD) using a microplate reader (Infinite 200, Tecan, Männedorf, Switzerland).

### Western blot

The method was performed essentially as detailed elsewhere [36]. Cell monolayers were harvested by scraping into lysis buffer (2% (w/v) SDS, 50 mM Tris-HCl, pH 7.4 supplemented with protease inhibitor [Minicomplete, Roche], 1mM Na_3_VO_4_, 1mM DTT) followed by 3 × 3 sec sonification (30% amplitude, Sonoplus HD 2070, Bandelin, Berlin, Germany) and centrifugation in a bench-top centrifuge (13.000 rpm, 10 min, 4°C). Supernatants were subjected to colorimetric quantification (Pierce BCA protein assay, Thermo Scientific) and stored at −20°C. SDS-PAGE gels were loaded with equal amounts of protein per lane (25 μg/lane) and transfer was visualized by Ponceau Red staining of nitrocellulose membranes. Primary and secondary peroxidase-coupled Abs (Amersham GE, Little Chalfont, UK) were diluted as recommended by the manufacturers. Chemoluminescence was detected using ECL (Amersham) and quantified in an automated luminescence imaging device (Fusion Solo, Peqlab VWR, Radnor, Pennsylvania).

### MALDI-MS imaging of lipids

Cells were transiently transfected with EV or MTMR7 plasmids for 48 h. Thereafter, cell monolayers were harvested by trypsinization, centrifuged and stored as pellets at −20°C. Lipid spectra were identified and quantified as published [37]. In brief, frozen cell pellets were cut into 10 μm sections using a Leica CM 1900 cryostat (Leica Biosystems, Nussloch, Germany) at −15°C and thaw-mounted in duplicates onto indium tin oxide-coated conductive glass slides (Bruker Daltonik GmbH, Bremen, Germany). 4-phenyl-α-cyanocinnamic acid amide (Ph-CCA-NH2) [5 mg/ml in acetone/water (90:10, v/v)] was deposited onto the cryosections using a matrix sprayer (SunChrom, Friedrichsdorf, Germany), where air pressure was set to 2.5 bar. Matrix was deposited in nine layers, with medium flow rate set to 10 μl/min (first layer); 15 μl/min (second); 20 μl/min (third) and 25 μl/min (all others). The z-position was set to 25.3 mm. MALDI-TOF MS was performed on an Autoflex III MALDI-TOF/TOF instrument (Bruker) equipped with a smartbeam laser (200 Hz) and controlled by flexControl 3.0 software (Bruker). The instrument was set to an acceleration voltage of 19 kV; spectra were acquired in reflector negative ion mode with baseline subtraction in the range from 400 to 1200 Da. Images were acquired at a spatial resolution of 100 μm with 200 laser shots per position by flexImaging 3.0 software (Bruker). The MALDI-MS/MS analysis using the LIFT cell of the Autoflex III instrument was conducted directly on the cell pellet sections after MALDI IMS in which parent ions were selected with a precursor ion selector window of 1% of their mass. The MALDI Imaging data were normalized to the total ion count and mass filters were chosen with a width of 0.2 Da.

### Immunohistochemistry (IHC)

Antibody (Ab) and haematoxylin and eosin (HE) stainings were done as described [35, 38]. In brief, tissue sections were pretreated in citrate buffer for antigen retrieval and incubated with hydrogen peroxide block and Ultra V Block (both Thermo Scientific, Braunschweig, Germany) to avoid unspecific reactions. IHC was performed using polyclonal rabbit anti-MTMR7 Ab (1:400, from Abcam). For visualization, the Histofine-HRP-Universal-Antibody Polymer (Medac, Wedel, Germany) and the 3,3′-diamino benzidine (DAB) substrate kit (VectorLabs, Peterborough, UK) were applied (brown color). Counterstaining was done with haematoxylin (Dr. K. Hollborn Söhne GmbH Co KG; Leipzig, Germany). The cytoplasmic staining was evaluated in the epithelial compartment (TU and NC) and the stroma (lamina propria) using a human smooth muscle biopsy as a positive control for calibration of the staining on each slide. The frequency and intensity of MTMR7 staining was assessed in human custom-made (from CR) and commercial (Co483, US Biomax, Rockville, MD) TMAs or in FFPE resection material and biopsies on slides (provided by JM, PK) using the following scores: 0+ = negative (0-25% positive rate), 1+ = weak (25-50%), 2+ = moderate (50-75%), 3+ = strong (75-100%). In addition, patient cases were analysed for dichotome distribution of staining positivity (0=negative *vs.* 1=positive). All analyses were done rater-blinded on a standard bright-field microscope using manual counting supported by Image J software (imagej.nih.gov/ij).

### ELISA

The PI(3)P (#K-3300) and PI(3,4,5)P3 (#K-2500) Mass ELISA Kits (both from Echelon Biosciences, Salt Lake City, UT) were used as recommended by the manufacturer. Acidic lipids were extracted from 5*10^6^ cells. OD values from serial dilutions of lipids (pure, 1:2, 1:4 and 1:8) were measured using a microplate reader (Infinite 200, Tecan) and calculated as pmol per 10^6^ cells from the standard curve using non-linear curve fitting software (Graphpad Prism 4.0).

### Reverse transcription PCR (RT-PCR) and quantitative PCR (qPCR)

Extraction of total RNA from cells and frozen tissues was carried out with peqGOLD Total RNA kit (VWR International, Darmstadt, Germany), and PCR methods were performed as published [36, 38]. Human *MTMR7* cDNA (NM_004686.4) was amplified with primer pairs against N-terminal (*N-MTMR7* 328-672), central/middle (*M-MTMR7* 1087-1220) and C-terminal (*C-MTMR7* 2087-2217) regions of the CDS. Sequences of oligonucleotides are listed in Supplementary Table S7. CT-values were normalized to the house-keeping gene beta2-microglobulin (*B2M, B2m*) and calculated as -fold induction using the formulas of the relative quantification ΔΔCT-method according to (https://lifescience.roche.com) [39].

### Calculation of optical density (OD)

Band intensities in gels from Western blots were quantified using Image J software (imagej.nih.gov/ij). OD values from proteins of interest were normalized to OD values of the corresponding house-keeping proteins (HSP90, beta-actin) or in case of phospho-epitopes to the unphosphorylated protein of interest. These normalized OD ratios from MTMR7-transfected cells were then substracted from the respective control cells transfected with empty vector (EV) for each time point or concentration. Statistical analysis was then performed on these differences (D = MTMR7-EV). OD values from colorimetric MTT assays were first normalized by division through the starting value, defined as 100% cell viability at day 1. Thereafter, values from MTMR7-transfected cells were divided through the respective EV-transfected control cells for each time point or concentration. Statistical analysis was then performed on these ratios (R = MTMR7/EV *vs.* EV/EV).

### Statistics and software

IHC data from patient tissue specimens were analysed with SPSS version 20.0 (IBM Corporation, Armonk, NY) as described [40, 41]. Univariate analyses were done to detect differences between patient groups using log-rank and Fisher exact tests regarding metabolic factors, tumor-specific parameters and survival. Median times and rates for survival were defined according to the NCI Dictionary of Cancer Terms (www.cancer.gov): OS rate = percentage of people who are still alive for a certain period of time (usually after a 5- or 10 year follow-up) after they were diagnosed for CRC. TSS rate = percentage of patients who have not died from the CRC in a defined period of time. The time period begins at the time of diagnosis and ends at the time of death. Patients who died from causes other than CRC were excluded from the measurement.

Quantitative results from cell experiments are expressed as means ± S.E. from at least 3 independent replicates from (i) the same cell line from different cultivation passages or (ii) from a pool of 3 different cell lines or stable clones as indicated in the result section and figure legends. Statistical analysis was performed using Graphpad Prism (version 4.0, La Jolla, CA). All tests were unpaired and two-sided. P-values (*p<0.05) were calculated using Student's t-test / Mann Whitney test for pair-wise comparisons and ANOVA / Kruskal-Wallis tests for multiple comparisons. Bioinformatic data were retrieved from cbioportal.org according to the TCGA publication guidelines [13, 14]. Chromosomal localization of genes was depicted with NCBI Mapviewer (ncbi.nlm.nih.gov/projects/mapview/).

## SUPPLEMENTARY MATERIALS FIGURES AND TABLES









## Abbreviations

Ab: antibody
AKT: protein kinase B
AMPK: AMP-activated protein kinase
B2M: beta-2-microglobulin
CC: coiled coil (domain)
CCLE: Cancer Cell Line Encyclopedia
CDS: coding sequence
CNA: copy number alteration
CRC: colorectal cancer
EGF: epidermal growth factor
ERK1/2: extracellular signal-regulated kinase 1/2
EV: empty vector
FFPE: formalin-fixed and paraffin-embedded
FL: full-length
G: tumor grade
IGF: insulin-like growth factor
IHC: immunohistochemistry
L: tumor invasion into lymph vessels
LOI: loss of imprinting
M: distant metastasis
MALDI-IMS: matrix assisted laser desorption/ionisation mass spectrometry imaging
MTM: myotubularin
MTMR: myotubularin-related protein
N: nodal spread (to lymphnodes)
NC: normal colon (tissue)
NTC: non-template (water) control
OD: optical density
OS: overall survival
PDK: 3-phosphoinositide-dependent protein kinase
PI: phosphatidylinositol
PI3K: PI3kinase
PIP: phosphoinositide
pT: pTarget (vector)
pTNM: tumor classification system
PTP: protein tyrosine phosphatase (domain)
RAS: rat sarcoma viral oncogene homolog
RTK: growth factor receptor tyrosine kinase
S6K: S6 protein kinase
S6RP: S6 ribosomal protein
SID: Set interaction domain
T: local tumor growth
T2DM: type 2 diabetes mellitus
TCGA: The Cancer Genome Atlas
TCL: total cell lysate
TMA: tissue microarray
mTORC1/2: mammalian target of rapamycin complex 1/2
TPA: 12-O-tetra decanoylphorbol-13-acetate
TSS: tumor-specific survival
TU: tumor (tissue)
UICC: union internationale contre le cancer
V: tumor invasion into veins.
