# Supplementary material for: Myotubularin-related protein 7 inhibits insulin signaling in colorectal cancer
Source: Oncotarget. 2016 Jul 7;7(31):50490–506. doi: 10.18632/oncotarget.10466 (PMC5226598; doi:10.18632/oncotarget.10466)
Supplement: Supplementary file 1 [file oncotarget-07-50490-s001.pdf]

Myotubularin-related protein 7 inhibits insulin signaling in colorectal cancer

SUPPLEMENTARY FIGURES AND TABLES

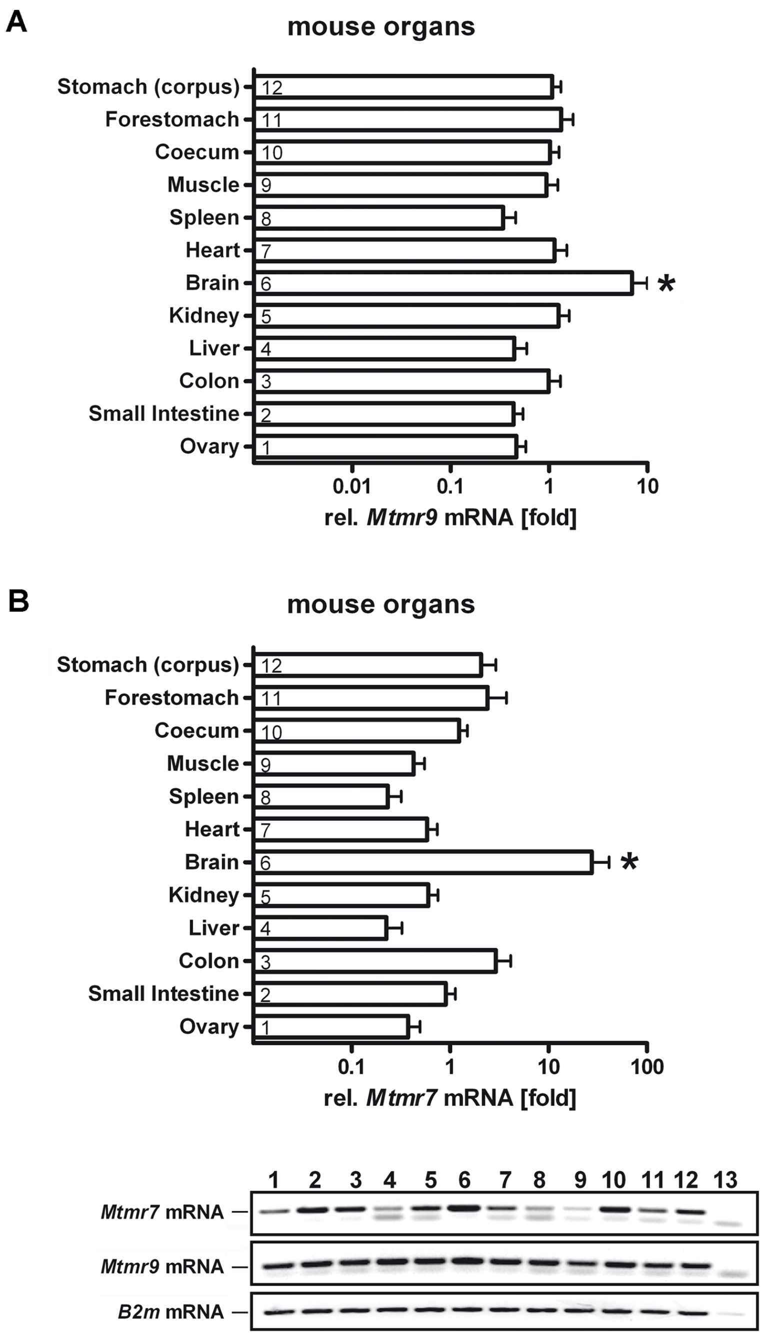

Supplementary Figure S1: Supportive RT-PCR analyses in mouse tissues. A.-B. Detection of *Mtmr7* and *Mtmr9* mRNAs in mouse organs. Representative agarose gels (endpoint 40 x PCR cycles) are shown together with quantitative analyses. CT-values from RT-qPCRs on total RNA were normalized to beta2-microglobulin (*B2m*) and calculated as -fold ± S.E. (n=3 mice; *p<0.05 *vs.* brain; Kruskal Wallis test). Legend: 1=Ovary, 2=Small intestine, 3=Colon, 4=Liver, 5=Kidney, 6=Brain, 7=Heart, 8=Spleen, 9=Skeletal muscle, 10=Coecum, 11=Forestomach, 12=Stomach (corpus), 13=NTC no template (water) control. Expected sizes of amplification products: *Mtmr7* = 172 bp, *Mtmr9* = 147 bp, *B2m* = 177 bp.

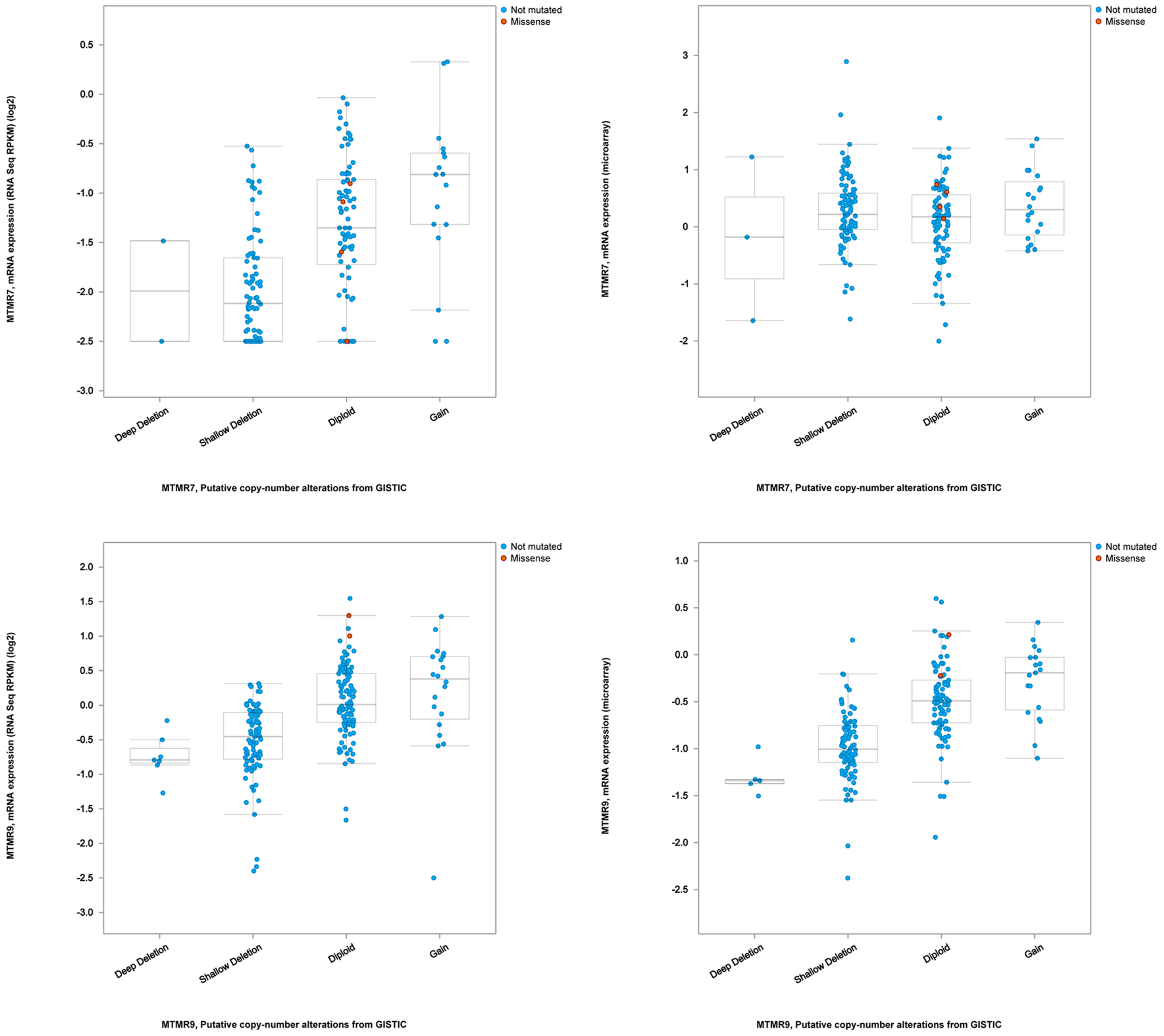

Supplementary Figure S2: Genomic alterations in *MTMR7* and *MTMR9* genes in a second cohort of CRC patients. Data were retrieved from the webportal cBioportal of Cancer Genomics based on the data set: colorectal carcinoma TCGA_Nature 2012 (n=195 cases). The mRNA expression (RNAseq V2 RSEM) (log2) is plotted against putative copy number alterations (CNA) from GISTIC.

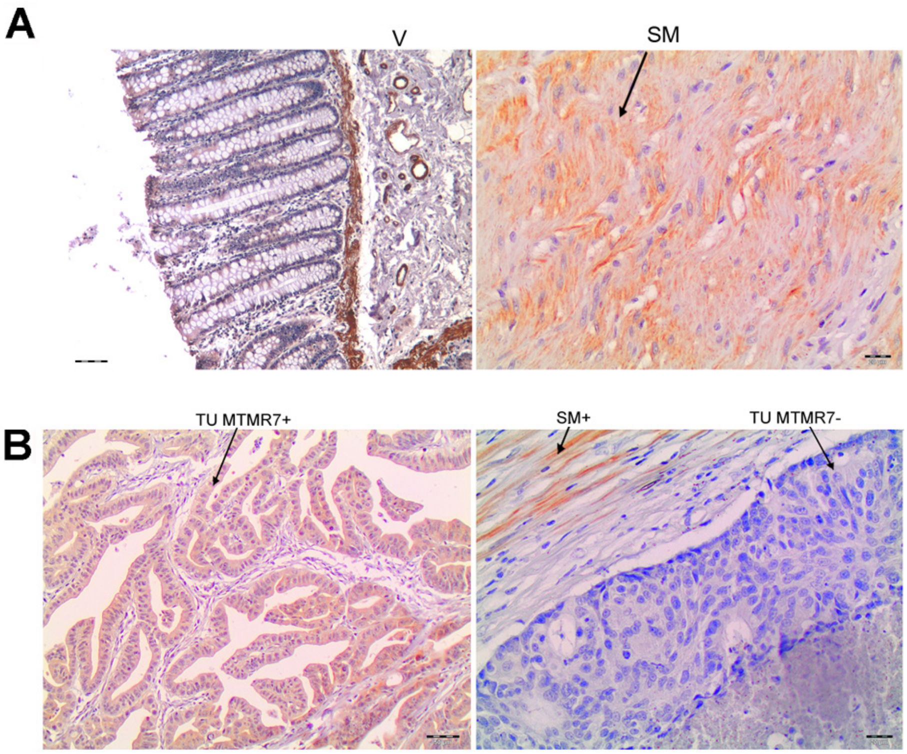

Supplementary Figure S3: Localization of MTMR7 protein in human normal and malignant colon. A. MTMR7 protein in benign human tissues. Representative microscopy images of IHC stainings detecting MTMR7 (Abcam Ab) in the epithelial (left half) and stromal (right half) compartments of the colon (left image) and in smooth muscle (right image). Note the weak cytoplasmic presence of MTMR7 in the normal (colonic crypt) epithelium (scale bar = 50 µm) and the strong positivity of smooth muscle (SM) staining around vessels (V) (scale bars = 20 µm). Original magnifications 200x, 400x. **B.** MTMR7 protein in human CRC. Representative IHC images detecting MTMR7 as in A. The staining of MTMR7 was present (left image) or lost (right image) in the tumor cells (scale bars = 50 and 20 µm). Note the still positive staining in smooth muscle cells adjacent to MTMR7 negative tumor areas. Original magnifications 200x, 400x.

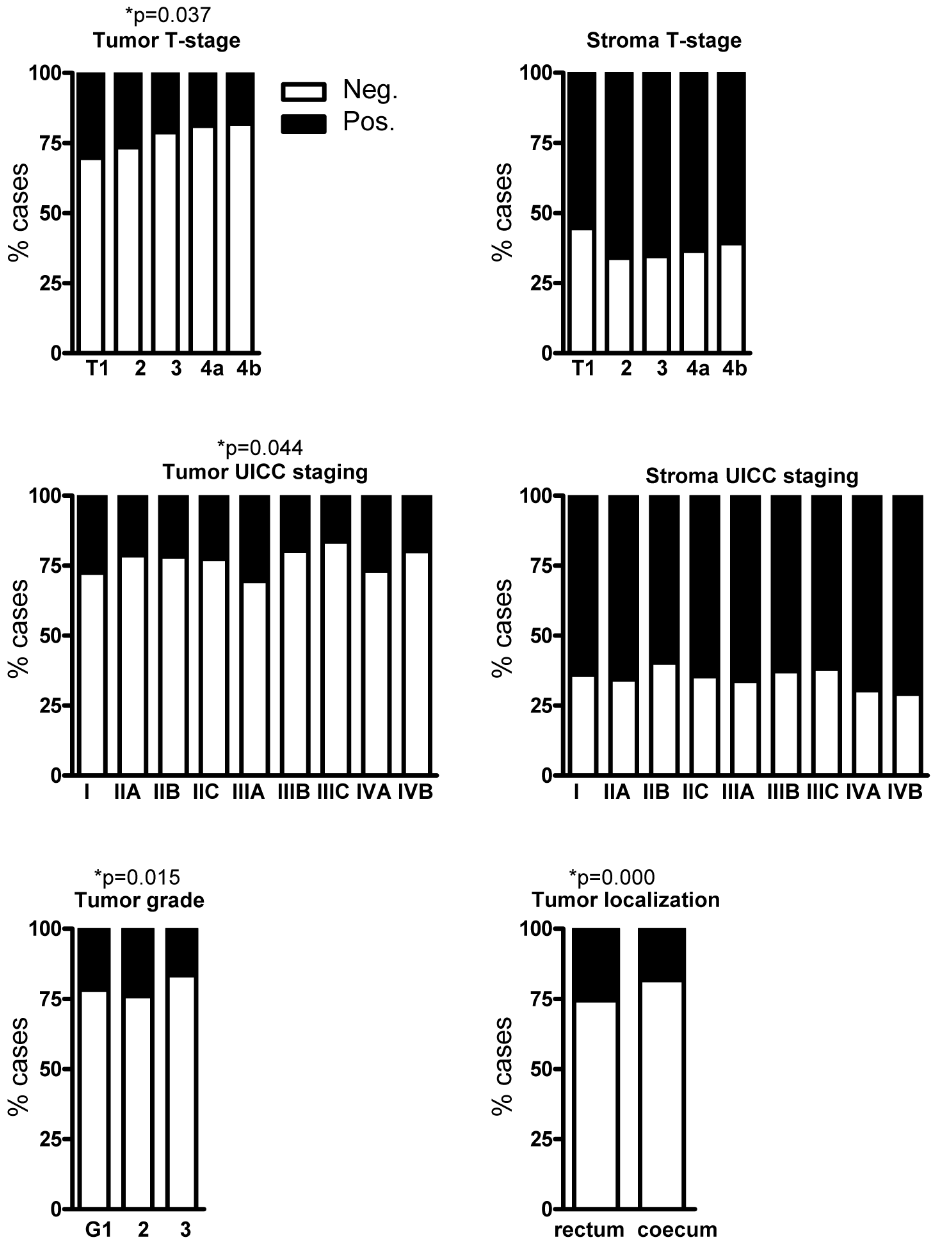

Supplementary Figure S4: Correlation of MTMR7 protein to clinical factors in CRC patients. IHC with MTMR7 Ab (from Abcam) on TMAs with mixed tumor-stroma (n=1776/1786) specimens from CRC patients. MTMR7 expression in tumor and stroma cells was correlated with clinical parameters: Significant differences are shown for tumor stage (UICC), local tumor growth (T), grade (G) and anatomic localization. Data are % MTMR7 positive *vs*. negative cases (*p<0.05; Fisher exact tests).

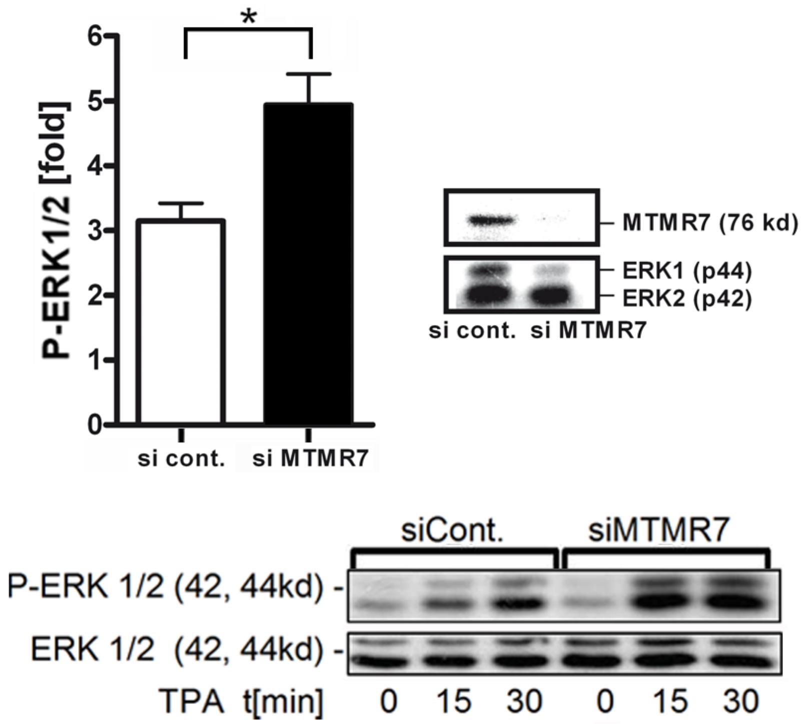

Supplementary Figure S5: MTMR7 knock-down enhances ERK1/2 phosphorylation. HCT116 cells were transiently transfected with MTMR7- or control-siRNA for 24 h, followed by serum-deprival for 24 h, and were then stimulated with the ERK1/2 pathway-activator TPA (100 nM) for 0 to 30 min. Representative Western blots and quantitative analyses are shown. OD values from P-ERK1/2 at time point 15 min were calculated as -fold ± S.E. of time point 0 in control cells (n=3; *p<0.05 MTMR7-siRNA *vs.* control-siRNA; t-test).

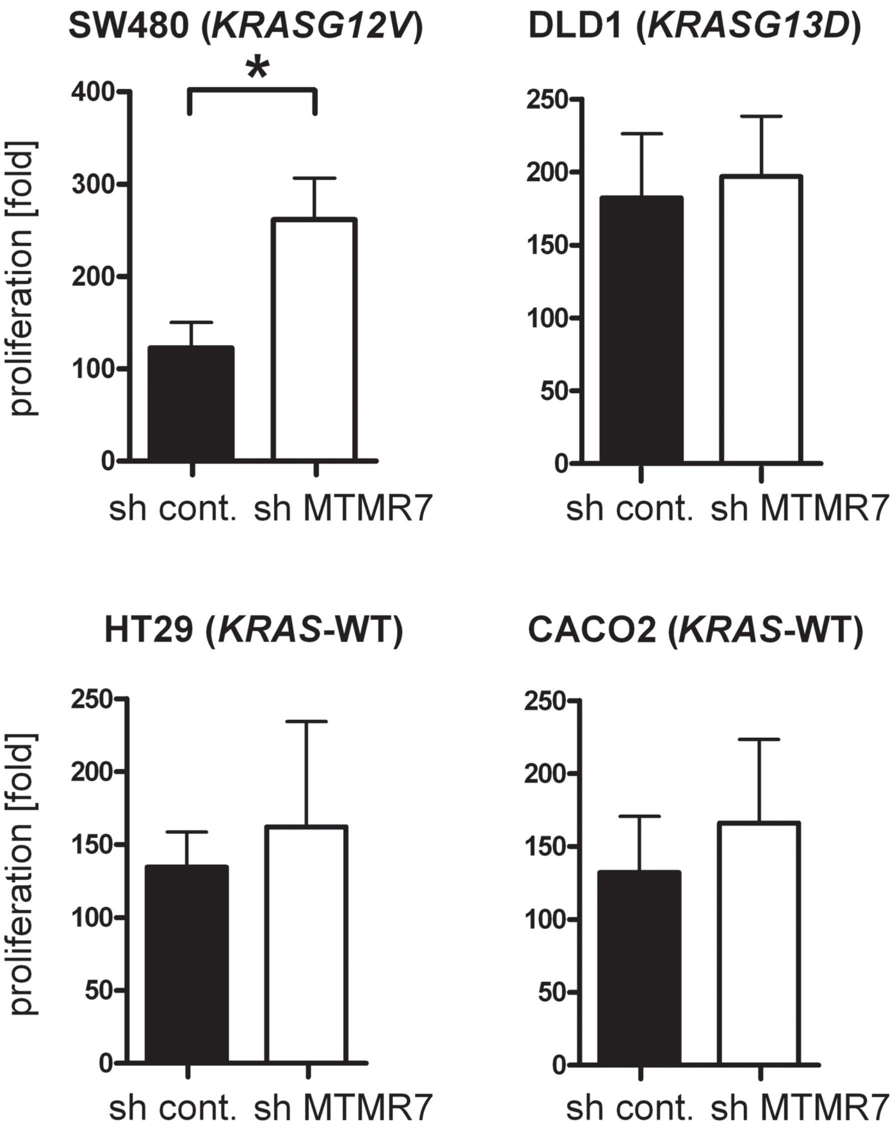

Supplementary Figure S6: MTMR7 knock-down accelerates proliferation. *KRAS* mutant (DLD1, SW480) and *KRAS*-WT (Caco2, HT29) cells were transiently transfected with MTMR7- or control-shRNA plasmid for 24 h, and proliferation was measured after 3 days. OD values were calculated as -fold ± S.E. (n=3; *p<0.05 MTMR7-shRNA *vs.* control-shRNA; Mann Whitney test) compared with day 0.

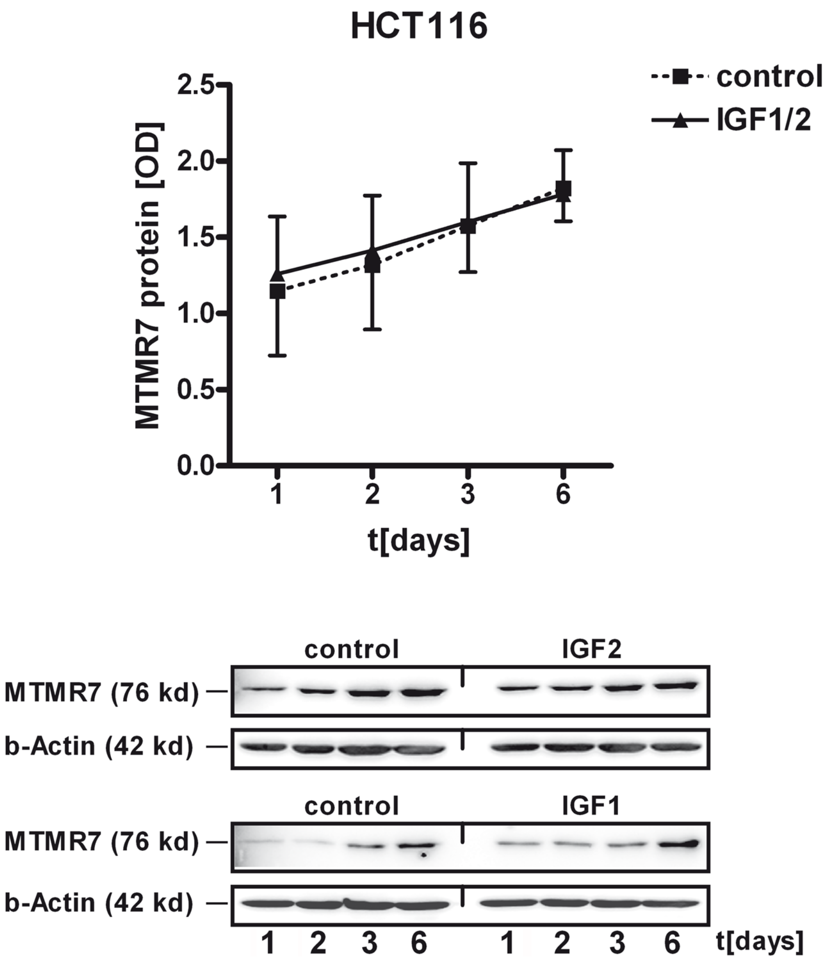

Supplementary Figure S7: Effect of IGFs on cellular MTMR7 protein. HCT116 cells were incubated without (control) or with IGF1/2 (at 50 ng/ml) in full medium for the days indicated, followed by Western blotting of TCLs. OD values from MTMR7 bands in gels were calculated as means ± S.E. of day 0 in control cells (n=3; n.s. treated *vs.* control; Kruskal Wallis test).

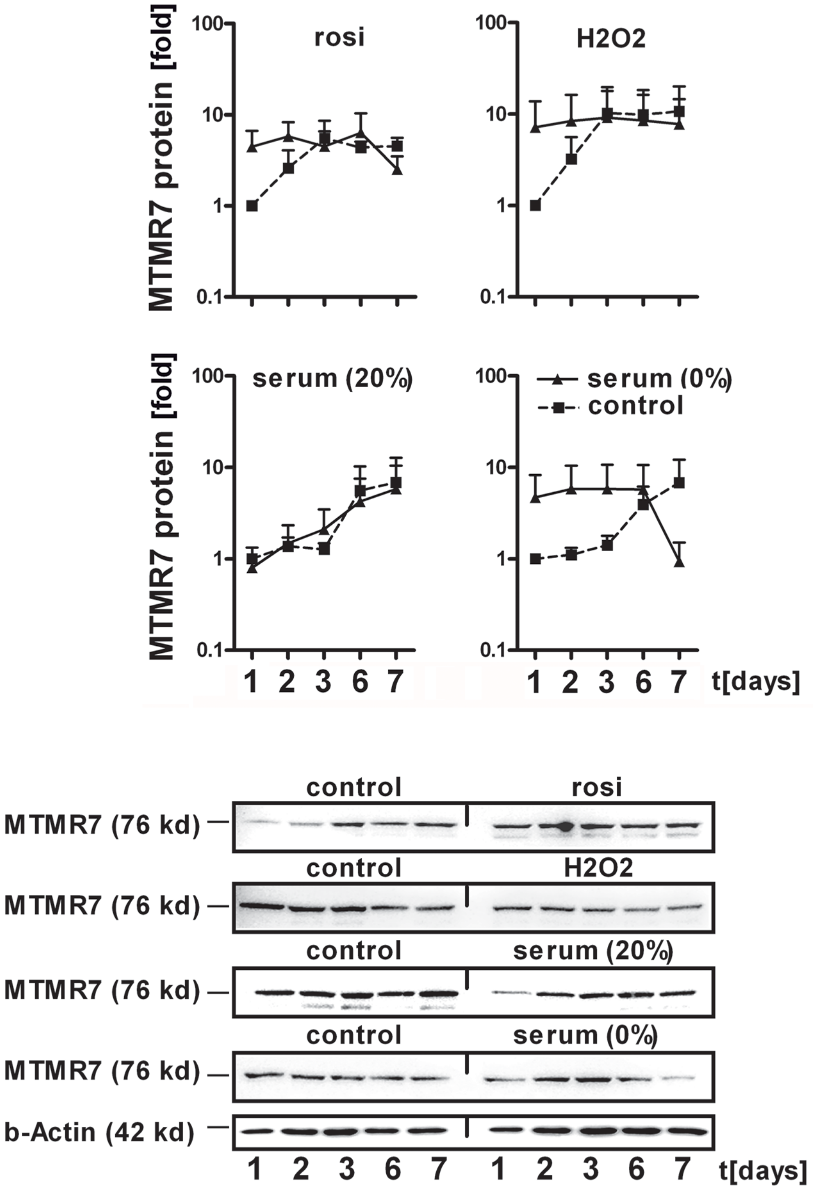

Supplementary Figure S8: Effect of other stimuli on cellular MTMR7 protein. HCT116 cells were incubated without (control) or with different stimuli [rosiglitazone (rosi) (1 µM), H_2O_2 (10 µM), serum (20% FCS)] in full medium or were cultivated in serum-free medium (0% FCS) for the days indicated, followed by Western blotting of TCLs. OD values from MTMR7 bands in gels were calculated as -fold ± S.E. of day 0 in control cells (n=3; n.s. treated *vs.* control; Kruskal Wallis test).

Supplementary Table S1: ***MTMR7*** **gene alterations in human cancers ***

Tumor data set	Cases altered	Type and number of alterations (N)				
		a	b	c	d	e
Colorectal Adenocarcinoma (TCGA, Provisional)	62 of 631 (10%)	1	40	5	16	0
Colorectal Adenocarcinoma (TCGA, Nature 2012)	11 of 195 (6%)	0	2	4	5	0
Liver Hepatocellular Carcinoma (TCGA, Provisional)	48 of 442 (11%)	2	33	4	9	0
Breast Invasive Carcinoma (TCGA, Nature 2012)	55 of 825 (7 %)	2	15	1	23	14
Breast Invasive Carcinoma (TCGA, Provisional)	96 of 1202 (8 %)	5	56	3	32	0
Breast Invasive Carcinoma (TCGA, Cell 2015)	96 of 1105 (9%)	5	56	3	32	0
Lung Adenocarcinoma (TCGA, Provisional)	43 of 522 (8%)	0	23	4	16	0
Pancreatic Adenocarcinoma (TCGA, Provisional)	15 of 186 (8 %)	1	2	1	11	0
Skin Cutaneous Melanoma (TCGA, Provisional)	53 of 478 (11 %)	1	9	14	29	0
Stomach Adenocarcinoma (TCGA, Nature 2014)	32 of 295 (11 %)	3	6	5	18	0
Stomach Adenocarcinoma (TCGA, Provisional)	25 of 443 (6 %)	9	6	5	4	1
Head and Neck Squamous Cell Carcinoma (TCGA, Provisional)	37 of 530 (7 %)	0	16	3	18	0
Head and Neck Squamous Cell Carcinoma (TCGA, Nature 2015)	22 of 279 (8 %)	0	9	3	10	0
Prostate Adenocarcinoma (TCGA, Provisional)	77 of 499 (15%)	0	66	1	10	0
Prostate Adenocarcinoma (TCGA, Cell 2015)	46 of 333 (14%)	0	41	1	4	0

* all tumors were included in the analysis. Legend: a = Amplification, b = Deep deletion, c = missense/truncation mutation, d = mRNA up-regulation, e = mRNA down-regulation.
N= number of patient cases.

Supplementary Table S2: *MTMR9* gene alterations in human cancers *

Tumor data set	Cases altered	Type and number of alterations (N)				
		**a**	**b**	**c**	**d**	**e**
Colorectal Adenocarcinoma (TCGA, Provisional)	64 of 631 (10%)	1	37	2	12	12
Colorectal Adenocarcinoma (TCGA, Nature 2012)	17 of 276 (6%)	0	2	7	4	4
Liver Hepatocellular Carcinoma (TCGA, Provisional)	110 of 442 (25%)	0	1	32	7	70
Breast Invasive Carcinoma (TCGA, Nature 2012)	61 of 825 (7%)	3	15	2	5	36
Breast Invasive Carcinoma (TCGA, Provisional)	156 of 1105 (14%)	2	62	3	89	0
Breast Invasive Carcinoma (TCGA, Cell 2015)	128 of 1105 (12%)	2	62	3	6	55
Lung Adenocarcinoma (TCGA, Provisional)	103 of 522 (20%)	2	25	3	22	51
Pancreatic Adenocarcinoma (TCGA, Provisional)	19 of 186 (10%)	5	1	1	8	4
Skin Cutaneous Melanoma (TCGA, Provisional)	42 of 478 (9%)	2	9	2	21	8
Stomach Adenocarcinoma (TCGA, Nature 2014)	45 of 295 (15%)	12	7	6	20	0
Stomach Adenocarcinoma (TCGA, Provisional)	42 of 443 (9%)	23	7	6	5	1
Head and Neck Squamous Cell Carcinoma (TCGA, Provisional)	43 of 530 (8%)	0	19	0	16	8
Head and Neck Squamous Cell Carcinoma (TCGA, Nature 2015)	37 of 279 (13%)	0	11	0	10	16
Prostate Adenocarcinoma (TCGA, Provisional)	81 of 499 (16%)	0	56	0	11	14
Prostate Adenocarcinoma (TCGA, Cell 2015)	52 of 333 (16%)	0	37	0	5	10

* all tumors were included in the analysis. Legend: a = Amplification, b = Deep deletion, c = missense/truncation mutation, d = mRNA up-regulation, e = mRNA down-regulation.
N= number of patient cases.

Supplementary Table S3: Correlation of MTMR7 expression with clinical factors in CRC patients.

See Supplementary File 1

Supplementary Table S4: Correlation of MTMR7 expression with prognosis (survival) in CRC patients.

See Supplementary File 2

Supplementary Table S5: *MTMR7* gene alterations and prognosis in human cancers *

Tumor data set	Gene alterations	Total cases	Cases deceased	Median months survival	p-value
Colorectal Adenocarcinoma (TCGA, Provisional)	yes	62	16	65.8	0.212 [OS]
	no	558	113	92.67	
	yes	51	15	NA	0.0592 [TSS]
	no	492	106	84.23	
Colorectal Adenocarcinoma (TCGA, Nature 2012)	yes	16	2	4.99	0.0883 [OS]
	no	258	24	NA	
	yes	No data	No data	No data	No data [TSS]
	no	No data	No data	No data	
Liver Hepatocellular Carcinoma (TCGA, Provisional)	yes	48	21	37.75	0.0298 [OS]
	no	328	111	69.51	
	yes	38	22	13.07	0.344 [TSS]
	no	287	157	21.16	
Pancreatic Adenocarcinoma (TCGA, Provisional)	yes	15	6	NA	0.0459 [OS]
	no	169	93	19.94	
	yes	12	4	NA	0.00311 [TSS]
	no	129	81	15.14	
Head and Neck Squamous Cell Carcinoma (TCGA, Provisional)	yes	37	10	NA	0.0349 [OS]
	no	488	213	53.91	
	yes	32	7	NA	0.0523 [TSS]
	no	364	138	61.07	

* all tumors were included in the analysis. Legend: OS = Overall survival, TSS = Tumor-specific survival, NA = not assessable (<50 % dead after >15 years), N= number of patient cases. # *MTMR9* gene alterations were n.s. (*p>=0.05) for all entities (data not shown).

Supplementary Table S6: Correlation of MTMR7 expression with clinical factors in CRC patients.

See Supplementary File 3

Supplementary Table S7: Primer sequences

Amplicon	F-primer	R-primer
N-MTMR7 (345 bp)	TGA CGG CTA CCC ATG TCA TA	AAT GAT TAG GGA GGC CCA TC
C-MTMR7 (131 bp)	TCT GTC AGC CAA CAG TGA CC	CAG TGA GAA ACA CGG CTT CA
M-MTMR7 (134 bp)	TGC AGA AAA TGC TGG AAG TG	CAC TGC CTT TGC AAT GAA GA
MTMR9 (174 bp)	CCA CCA CTT GAT CCT GTC CT	TTC AAG CAT TCC TCC ATT CC
B2M (85 bp)	TGC TGT CTC CAT GTT TGA TGT ATC T	TCT CTG CTC CCC ACC TCT AAG T
Mtmr7 (172 bp)	CTG CAG GGA AAG GCT ATG AG	CAG CCT GAG TTC TCC AGT CC
Mtmr9 (147 bp)	CGA AGC ACT TCG GAA GGT AG	TTC TCG TCT TCC TTG CAC CT
B2m (177 bp)	ATG GGA AGC CGA ACA TAC TG	CAG TCT CAG TGG GGG TGA AT
