# Supplementary material for: Myotubularin-related protein 7 inhibits insulin signaling in colorectal cancer
Source: Oncotarget. 2016 Jul 7;7(31):50490–506. doi: 10.18632/oncotarget.10466 (PMC5226598; doi:10.18632/oncotarget.10466)
Supplement: Supplementary file 2 [file oncotarget-07-50490-s002.doc]

| **Table S3 Correlation of MTMR7 expression with clinical factors in CRC patients** | | | | |
| --- | --- | --- | --- | --- |
|  | **Tumor** | | **Stroma** | |
| **Total** | **Positive** | **Negative** | **Positive** | **Negative** |
|  | 399/1776 [22.5%] | 1377/1776 [77.5%] | 1154/1786 [64.6%] | 632/1786 [35.4%] |
| **Gender** |  | |  | |
| Male | 213/915 [23.3%] | 702/915 [76.7%] | 608/921 [66.0%] | 313/921 [34.0%] |
| Female | 186/861 [21.6%] | 675/861 [78.4%] | 546/865 [63.1%] | 319/865 [36.9%] |
| **Age** |  | |  | |
| <71 | 198/840 [23.6%] | 642/840 [76.4%] | 557/847 [65.8%] | 290/847 [34.2%] |
| ≥71 | 191/877 [21.8%] | 686/877 [78.2%] | 562/880 [63.9%] | 318/880 [36.1%] |
| **Localization** | **p≤0.0001** | | **p≤0.0001** | |
| Rectum – Sigma | 239/937 [25.5%] | 698/937 [74.5%] | 652/946 [68.9%] | 294/946 [31.1%] |
| C.desc. – Coecum | 134/732 [18.3%] | 598/732 [81.7%] | 431/730 [59.0%] | 299/730 [41.0%] |
| **Staging** |  | |  | |
| **T** | **p=0.037** | |  | |
| T1 | 27/89 [30.3%] | 62/89 [69.7%] | 51/92 [55.4%] | 41/92 [44.6%] |
| T2 | 105/396 [26.5%] | 291/396 [73.5%] | 264/400 [66.0%] | 136/400 [34.0%] |
| T3 | 211/998 [21.1%] | 787/998 [78.9%] | 655/1002 [65.4%] | 347/1002 [34.6%] |
| T4a | 38/201 [18.9%] | 163/201 [81.1%] | 127/200 [63.5%] | 73/200 [36.5%] |
| T4b | 16/89 [18.0%] | 73/89 [82.0%] | 54/89 [60.7%] | 35/89 [39.3%] |
| **T 1/2 vs 3/4** | **p=0.003** | |  | |
| T1/T2 | 132/485 [27.2%] | 353/485 [72.8%] | 315/492 [64.0%] | 177/492 [36.0%] |
| T3/T4 | 265/1288 [20.6%] | 1023/1288 [79.4%] | 836/1291 [64.8%] | 455/1291 [35.2%] |
| **N** |  | |  | |
| N0 | 234/978 [23.9%] | 744/978 [76.1%] | 639/986 [64.8%] | 347/986 [35.2%] |
| N1a | 38/170 [22.4%] | 132/170 [77.6%] | 97/171 [56.7%] | 74/171 [43.3%] |
| N1b | 39/186 [21.0%] | 147/186 [79.0%] | 132/188 [70.2%] | 56/188 [29.8%] |
| N1c | 1/3 [33.3%] | 2/3 [66.7%] | 2/3 [66.7%] | 1/3 [33.3%] |
| N2a | 42/187 [22.5%] | 145/187 [77.5%] | 127/186 [68.3%] | 59/186 [31.7%] |
| N2b | 37/228 [16.2%] | 191/228 [83.8%] | 140/228 [61.4%] | 88/228 [38.6%] |
| **M** |  | |  | |
| M1 | 50/205 [24.4%] | 155/205 [75.6%] | 144/204 [70.6%] | 60/204 [29.4%] |
| Mx | 348/1569 [22.2%] | 1221/1569 [77.8%] | 1008/1580 [63.8%] | 572/1580 [36.2%] |
| **L** | **p=0.056** | |  | |
| L0 | 314/1335 [23.5%] | 1021/1335 [76.5%] | 867/1345 [64.5%] | 478/1345 [35.5%] |
| L1 | 84/439 [19.1%] | 355/439 [80.9%] | 285/439 [64.9%] | 154/439 [35.1%] |
| **V** |  | |  | |
| V0 | 368/1649 [22.3%] | 1281/1649 [77.7%] | 1063/1657 [64.2%] | 594/1657 [35.8%] |
| V1 | 30/125 [24.0%] | 95/125 [76.0%] | 89/127 [70.1%] | 38/127 [29.9%] |
| **UICC Stage** | **p=0.044** | |  | |
| I | 106/385 [27.5%] | 279/385 [72.5%] | 251/392 [64.0%] | 141/392 [36.0%] |
| IIa | 105/493 [21.3%] | 388/493 [78.7%] | 324/494 [65.6%] | 170/494 [34.4%] |
| IIb | 10/46 [21.7%] | 36/46 [78.3%] | 28/47 [59.6%] | 19/47 [40.4%] |
| IIc | 7/31 [22.6%] | 24/31 [77.4%] | 20/31 [64.5%] | 11/31 [35.5%] |
| IIIa | 18/59 [30.5%] | 41/59 [69.5%] | 39/59 [66.1%] | 20/59 [33.9%] |
| IIIb | 69/352 [19.6%] | 283/352 [80.4%] | 222/354 [62.7%] | 132/354 [37.3%] |
| IIIc | 33/201 [16.4%] | 168/201 [83.6%] | 124/201 [61.7%] | 77/201 [38.3%] |
| IVa | 35/131 [26.7%] | 96/131 [73.3%] | 91/131 [69.5%] | 40/131 [30.5%] |
| IVb | 15/76 [19.7%] | 61/76 [80.3%] | 53/75 [70.7%] | 22/75 [29.3%] |
| **UICC Stage I-IV** | **p=0.027** | |  | |
| I | 106/385 [27.5%] | 279/385 [72.5%] | 251/392 [64.0%] | 141/392 [36.0%] |
| II | 122/570 [21.4%] | 448/570 [78.6%] | 372/572 [65.0%] | 200/572 [35.0%] |
| III | 120/612 [19.6%] | 492/612 [80.4%] | 385/614 [62.7%] | 229/614 [37.3%] |
| IV | 50/207 [24.2%] | 157/207 [75.8%] | 144/206 [69.9%] | 62/206 [30.1%] |
| **Grading** | **p=0.015** | |  | |
| G1 | 12/55 [21.8%] | 43/55 [78.2%] | 38/54 [70.4%] | 16/54 [29.6%] |
| G2 | 324/1349 [24.0%] | 1025/1349 [76.0%] | 873/1358 [64.3%] | 485/1358 [35.7%] |
| G3 | 53/319 [16.6%] | 266/319 [83.4%] | 195/319 [61.1%] | 124/319 [38.9%] |
