# Supplementary material for: Myotubularin-related protein 7 inhibits insulin signaling in colorectal cancer
Source: Oncotarget. 2016 Jul 7;7(31):50490–506. doi: 10.18632/oncotarget.10466 (PMC5226598; doi:10.18632/oncotarget.10466)

| **Table S4 Association of MTMR7 with prognosis in CRC patients by UICC stage ***  * case numbers: Stage I (n=346); II (n=529); III (n=551), IV (n=184) | | | | | | | |
| --- | --- | --- | --- | --- | --- | --- | --- |
| **Median OS ± S.E. (months)** | | | | **5-Year OS (%)** | | **10-Year OS (%)** | |
| **MTMR7 expression** | **Log-rank (Mantel-Cox)** | **Negative** | **Positive** | **Negative** | **Positive** | **Negative** | **Positive** |
| **Tumor** |  |  |  |  |  |  |  |
| **Stage I** | **p=0.726** | >200  (n=244) | >200  (n=93) | 80.5 ± 2.6 | 77.4 ± 4.3 | 71.4 ± 3.2 | 71.7 ± 5.4 |
| **Stage II a/b/c** | **p=0.363** | >200  (n=412) | >200  (n=114) | 68.9 ± 2.3 | 60.3 ± 4.6 | 55.5 ± 2.8 | 51.8 ± 5.2 |
| **Stage III a/b/c** | **p=0.405** | 39.62 ± 6.8 [CI 95% 26.31-52.93] (n=441) | 51.35 ± 21.2 [CI 95% 9.80- 92.90] (n=108) | 44.6 ± 2.4 | 48.1 ± 4.8 | 36.5 ± 2.5 | 40.6 ± 5.3 |
| **Stage IV a/b/c** | **p=0.922** | 20.70 ± 3.7 [CI 95% 13.43-27.97] (n=136) | 19.06 ± 3.7  [CI 95% 11.86-26.25] (n=47) | 20.4 ± 3.6 | 17.0 ± 5.5 | 12.1 ± 3.6 | 17.0 ± 5.5 |
| **Stroma** |  |  |  |  |  |  |  |
| **Stage I** | ***p=0.032** | >200  (n=122) | >200  (n=223) | 87.6 ± 3.0 | 75.6 ± 2.9 | 78.0 ± 4.2 | 68.8 ± 3.4 |
| **Stage II a/b/c** | **p=0.208** | >200  (n=181) | >200  (n=346) | 63.3 ± 3.6 | 68.7 ± 2.5 | 51.8 ± 4.1 | 55.4 ± 3.1 |
| **Stage III a/b/c** | **p=0.729** | 39.89 ± 9.3  [CI 95% 21.57-58.20] (n=204) | 42.55 ± 7.3 [CI 95% 28.24-56.85] (n=347) | 44.7 ± 3.5 | 45.0 ± 2.7 | 34.0 ± 3.7 | 38.7 ± 2.8 |
| **Stage IV a/b/c** | **p=0.346** | 20.70 ± 5.7  [CI 95% 9.61- 31.78] (n=55) | 20.67 ± 2.8  [CI 95% 15.25-26.08] (n=127) | 15.9 ± 5.0 | 22.0 ± 3.8 | 8.0 ± 4.7 | 17.1 ± 3.9 |
| **Median TSS ± S.E. (months)** | | | | **5-Year TSS (%)** | | **10-Year TSS (%)** | |
| **MTMR7 expression** | **Log-rank (Mantel-Cox)** | **Negative** | **Positive** | **Negative** | **Positive** | **Negative** | **Positive** |
| **Tumor** |  |  |  |  |  |  |  |
| **Stage I** | **p=0.351** | >200  (n=244) | >200  (n=93) | 91.9 ± 1.8 | 86.5 ± 3.6 | 88.9 ± 2.3 | 86.5 ± 3.6 |
| **Stage II a/b/c** | **p=0.984** | >200  (n=408) | >200  (n=113) | 80.8 ± 2.0 | 76.8 ± 4.2 | 74.6 ± 2.5 | 76.8 ± 4.2 |
| **Stage III a/b/c** | **p=0.537** | 83.52 (n=432) | >200 (n=106) | 54.0 ± 2.5 | 55.0 ± 5.0 | 48.5 ± 2.7 | 55.0 ± 5.0 |
| **Stage IV a/b/c** | **p=0.977** | 21.32 ± 3.5  [CI 95% 14.51-28.14] (n=134) | 20.67 ± 3.8  [CI 95% 13.28-28.05] (n=45) | 22.6 ± 3.8 | 21.5 ± 6.2 | 16.3 ± 4.2 | 21.5 ± 6.2 |
| **Stroma** |  |  |  |  |  |  |  |
| **Stage I** | ***p=0.032** | >200  (n=122) | >200  (n=223) | 95.8 ± 1.8 | 87.7 ± 2.3 | 92.9 ± 2.7 | 86.0 ± 2.5 |
| **Stage II a/b/c** | **p=0.278** | >200  (n=180) | >200  (n=342) | 75.8 ± 3.3 | 82.2 ± 2.2 | 73.8 ± 3.5 | 75.7 ± 2.8 |
| **Stage III a/b/c** | **p=0.753** | 119.59 (n=198) | 83.48 (n=341) | 55.0 ± 3.7 | 53.5 ± 2.8 | 48.6 ± 4.2 | 50.0 ± 2.9 |
| **Stage IV a/b/c** | **p=0.303** | 20.70 ± 6.1  [CI 95% 8.75- 32.65] (n=54) | 22.01 ± 2.4  [CI 95% 17.38-26.64] (n=124) | 17.6 ± 5.4 | 25.1 ± 4.1 | 11.7 ± 6.0 | 21.3 ± 4.3 |


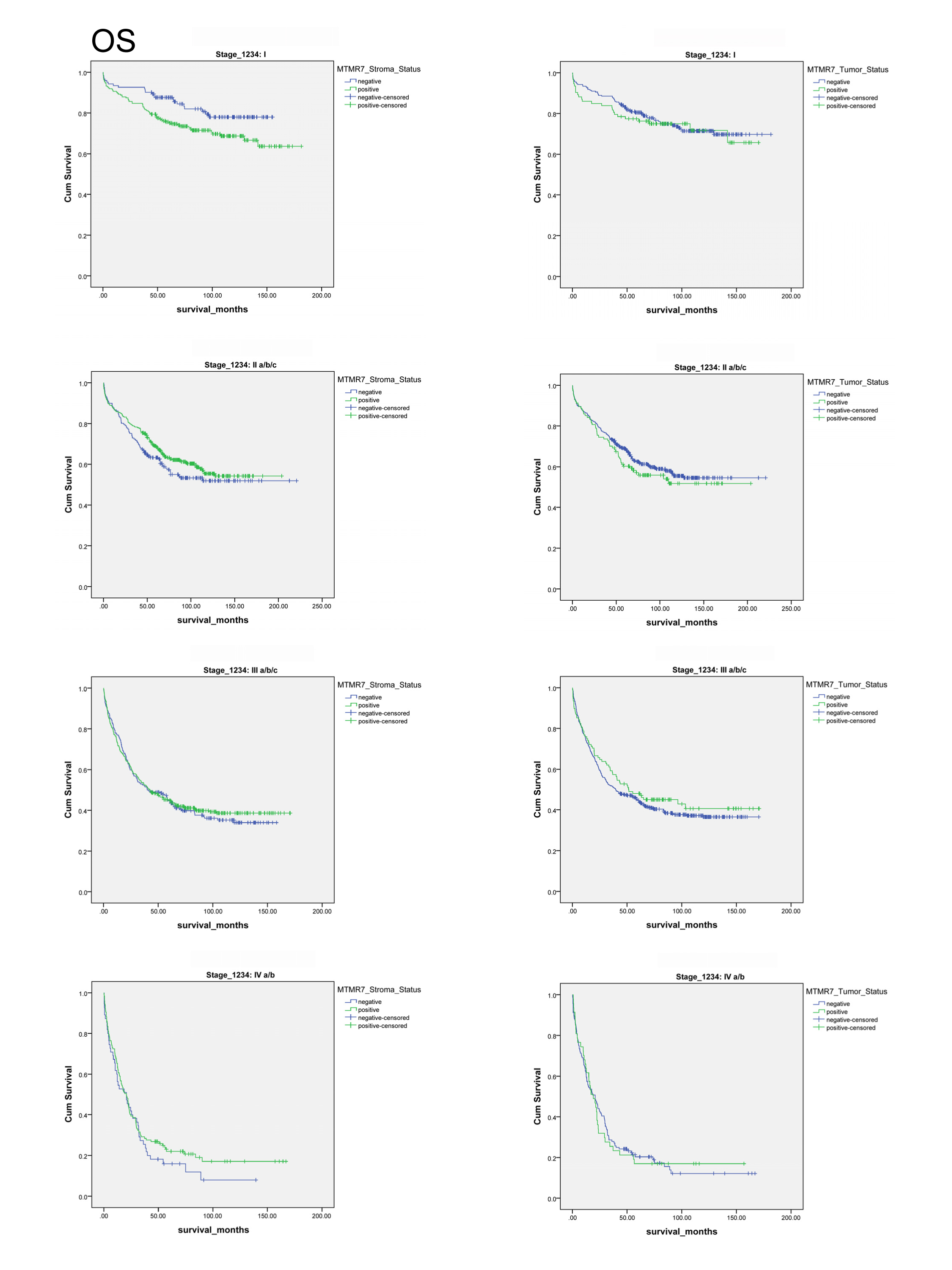


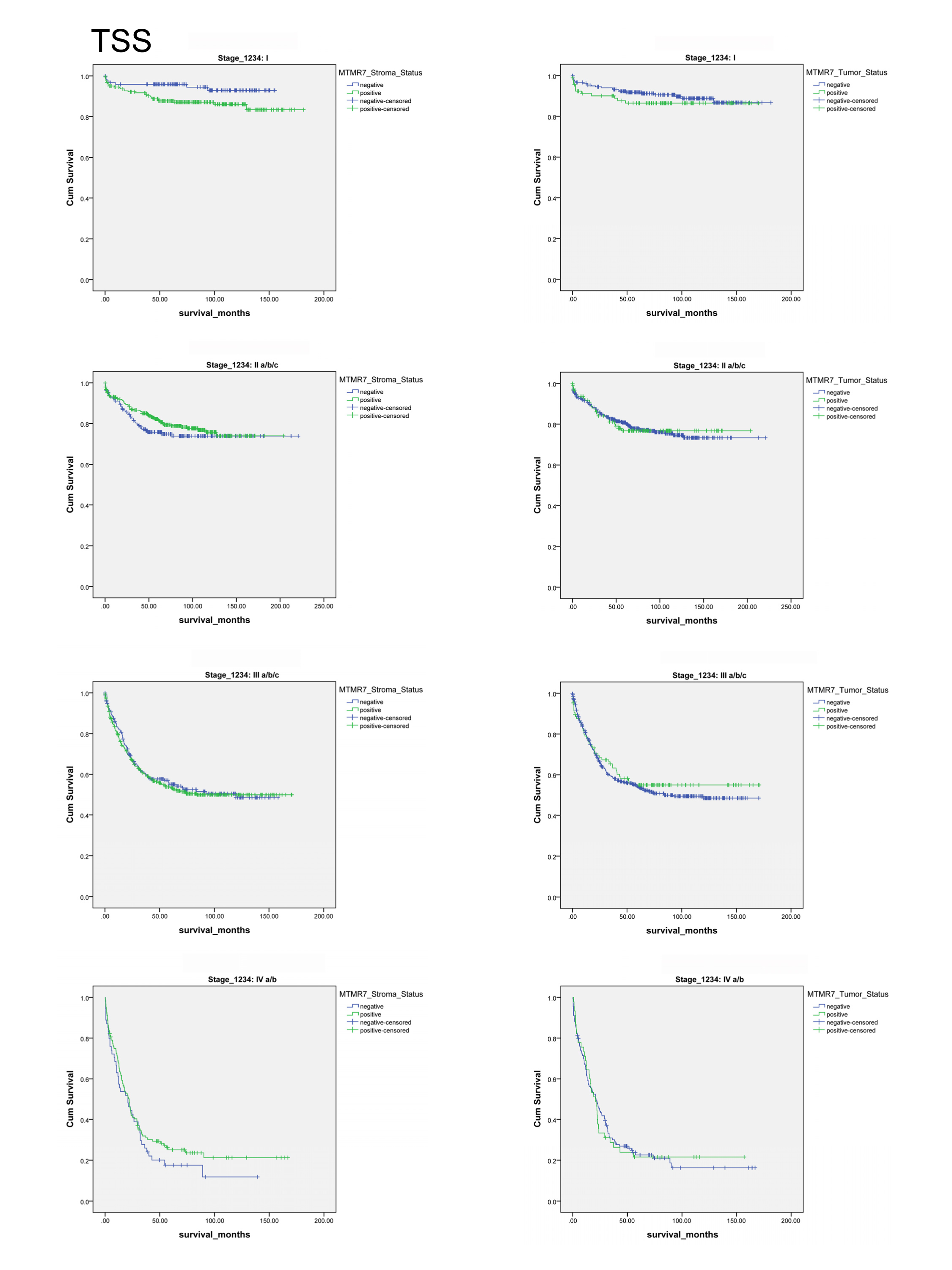

Supplement: Supplementary file 3 [file oncotarget-07-50490-s003.doc]
