# Supplementary material for: Myotubularin-related protein 7 inhibits insulin signaling in colorectal cancer
Source: Oncotarget. 2016 Jul 7;7(31):50490–506. doi: 10.18632/oncotarget.10466 (PMC5226598; doi:10.18632/oncotarget.10466)
Supplement: Supplementary file 4 [file oncotarget-07-50490-s004.doc]

| **Table S6 Correlation of MTMR7 expression with clinical factors in CRC patients** | | | | | |
| --- | --- | --- | --- | --- | --- |
| **MTMR7 cytoplasmatic staining** | | | | | |
|  | **positive** | | **Negative** | | **total** |
|  | **No.** | **%** | **No.** | **%** | **No.** |
| normal colon mucosa | 17 | 100.0 | 0 | 00.00 | 17 |
| colorectal cancer tissue | 51 | 45.13 | 62 | 54.87 | 113 |
| **Age** | | | | | |
| < 50 | 10 | 52.63 | 9 | 47.37 | 19 |
| 50-65 | 17 | 50.00 | 17 | 50.00 | 34 |
| >65 | 24 | 45.28 | 29 | 54.72 | 53 |
| **Gender** | | | | | |
| Male | 33 | 50.00 | 33 | 50.00 | 66 |
| Female | 16 | 48.48 | 17 | 51.52 | 33 |
| **Localization** | | | | | |
| Cecum | 1 | 25.00 | 3 | 75.00 | 4 |
| Ascending & right flexur | 3 | 42.86 | 4 | 57.14 | 7 |
| Transverse | 1 | 20.00 | 4 | 80.00 | 5 |
| Descending | 0 | 00.00 | 1 | 100.0 | 1 |
| Sigma | 7 | 50.00 | 7 | 50.00 | 14 |
| Rectum | 11 | 50.00 | 11 | 50.00 | 22 |
| **UICC Stage** | | | | | |
| ***1*** | 3 | 25.00 | 9 | 75.00 | 12 |
| ***2*** | 10 | 43.48 | 13 | 56.52 | 23 |
| 2A | 5 | 35.71 | 9 | 64.29 | 14 |
| 2B | 5 | 55.56 | 4 | 44.44 | 9 |
| ***3*** | 8 | 32.00 | 17 | 68.00 | 25 |
| 3A | 0 | 00.00 | 2 | 100.0 | 2 |
| 3B | 6 | 33.33 | 12 | 66.67 | 18 |
| **3C** | 2 | 50.00 | 2 | 50.00 | 4 |
| ***4*** | 2 | 18.18 | 9 | 81.82 | 11 |
| 4A | 2 | 28.57 | 5 | 71.43 | 7 |
| 4B | 0 | 00.00 | 2 | 100.0 | 2 |
| **Staging** | | | | | |
| **T** | | | | | |
| 1 | 3 | 100.0 | 0 | 00.00 | 3 |
| 2 | 9 | 39.13 | 14 | 60.87 | 23 |
| 3 | 24 | 44.44 | 30 | 55.56 | 54 |
| 4 | 15 | 48.39 | 16 | 51.61 | 31 |
| **N** | | | | | |
| N0 | 30 | 50.00 | 30 | 50.00 | 60 |
| N1 | 12 | 40.00 | 18 | 60.00 | 30 |
| N2 | 7 | 41.18 | 10 | 58.82 | 17 |
| **M** | | | | | |
| 0 | 23 | 37.10 | 39 | 62.90 | 62 |
| 1 | 6 | 35.30 | 11 | 64.71 | 17 |
| **L** | | | | | |
| 0 | 7 | 50.00 | 7 | 50.00 | 14 |
| 1 | 5 | 26.32 | 14 | 73.68 | 19 |
| **V** | | | | | |
| 0 | 27 | 58.70 | 19 | 41.30 | 46 |
| 1 | 7 | 50.00 | 7 | 50.00 | 14 |
| **Grading** | | | | | |
| G1 | 7 | 63.64 | 4 | 36.36 | 11 |
| G2 | 33 | 47.14 | 37 | 52.86 | 70 |
| G3 | 8 | 32.00 | 17 | 68.00 | 25 |
| **T2DM & *IGF2* LOI** | | | | | |
| No T2DM &  No *IGF2* LOI (control) | 9 | 56.25 | 7 | 43.75 | 16 |
| Yes  T2DM | 3 | 15.00 | 17 | 85.00 | 20 |
| YesT2DM &  No *IGF2* LOI | 2 | 11.76 | 15 | 88.24 | 17 |
| Yes *IGF2* LOI &  No T2DM | 1 | 07.69 | 12 | 92.31 | 13 |
